# Preliminary exploration of the relationship between ginsenoside content and endogenous hormones of multi stem ginseng and soil properties based on correlation analysis

**DOI:** 10.3389/fpls.2025.1706388

**Published:** 2025-11-17

**Authors:** Yuqiu Chen, Zhefeng Xu, Weichen Qi, Tao Zhang, Changbao Chen, Xiaoling Shang

**Affiliations:** 1 Basic Medical School, Changchun University of Chinese Medicine, Changchun, China; 2 Jilin Ginseng Academy, Changchun University of Chinese Medicine, Changchun, China; 3 College of Pharmacy, Changchun University of Chinese Medicine, Changchun, China

**Keywords:** multi stem ginseng, ginsenoside, endogenous hormones, soil abiotic factors, soil enzyme activities

## Abstract

**Aims:**

Compared with single stem ginseng (G1), there is a significant gap in the research on the morphology and quality formation of multi stem ginseng (MG).

**Methods:**

This study measured the ginsenoside content in the roots, stems, and leaves of 3-year-old single stem ginseng, double stem ginseng (G2), and triple stem ginseng (G3), as well as the endogenous hormone content in the rhizomes. At the same time, the physicochemical properties and enzyme activity of the rhizosphere and non-rhizosphere soil were measured, and the differences and connections between each indicator were analyzed.

**Results:**

ResultsThe content of ginsenosides in the roots of MG was significantly higher than that in G1, with the total ginsenoside content in G2 and G3 being 1.30 and 1.24 times higher than that in G1. There was a significant difference in the content of salicylic acid and brassinolide between G1 and MG. Differential analysis showed that aluminum bound phosphorus, aggregates with a particle size of 2-3 mm, and praseodymium were characteristic abiotic factors that contributed to differences in soil properties, fluorescein diacetate, peroxidase and invertase were characteristic enzymes that differed in different soils. A regulatory network of "soil abiotic factor-ginsenosides-soil enzymes" was constructed based on the results of correlation analysis.

**Conclusions:**

ConclusionsThe interaction between soil enzymes (fluorescein diacetate, peroxidase, ascorbate peroxidase) and abiotic factors (praseodymium, neodymium, yttrium) were the main influencing factors of ginsenoside accumulation in MG. The results had improved our understanding and helped to better guide the planting of MG.

## Graphical Abstract

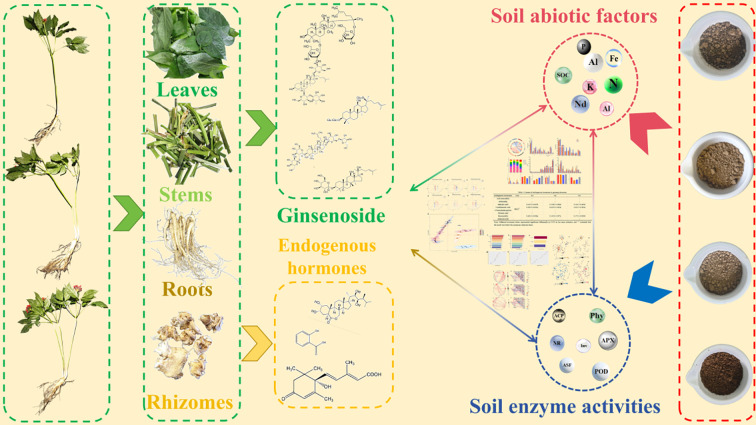

## 1 Introduction

Ginseng (*Panax ginseng* C. A. Mey) is a traditional Chinese herbal medicine with great medicinal value. It is mainly produced in the north latitude range of 40-44° and the east longitude range of 117.5-134°. The temperate continental monsoon climate is a unique climatic ecological type formed by high-quality ginseng (Dong et al., 2022; Baeg and So, 2013). China, Japan, South Korea, Russia are the main producing areas of ginseng. In China, ginseng mainly grows in the northeast region (Yu et al., 2017). The high medicinal value of ginseng can be explained by its rich and diverse chemical composition. Ginsenosides, polysaccharides, proteins, volatile oils, peptides, and amino acids are the main chemical components in ginseng roots, all of which have different pharmacological activities. Among them, ginsenosides are the most active component of ginseng pharmacological activity (Chen et al., 2019; Ratan et al., 2021; Liu et al., 2021). Therefore, the study of the composition and content of ginsenosides has always been a hot topic of concern for researchers.

Cool, mild climate conditions and special soil conditions are essential for the production of high-quality ginseng. Forest soil is the most ideal environment for ginseng growth, characterized by abundant organic matter content, good air and water permeability, slight acidity, and balanced nutrient structure (You et al., 2015). For the purpose of sustainable development and protection of forest resources, large-scale forest planting of ginseng is no longer allowed, and in order to meet the demand of the ginseng market, it is necessary to vigorously develop agricultural ginseng planting models (Yu et al., 2020). Due to unreasonable fertilization patterns, the quality of farmland soil is often uneven and the nutrient structure is unbalanced, which leads to many problems in the cultivation of ginseng. Therefore, research on high-quality and sustainable farmland ginseng cultivation is a long-term direction that needs to be studied. Soil enzymes refer to biologically active proteins present in soil, mainly derived from the secretion of soil microorganisms. Soil enzyme activity sensitively reflects soil microbial activity (Saleem and Ahmed, 2020). In addition, soil enzyme activity also indicates many biochemical processes, such as soil humus decomposition, organic matter transformation, and the supply of available nutrients, which plays a critical role in the material cycle and energy flow of ecosystems (Yao et al., 2022). Therefore, the study of soil enzymes has essential practical significance.

Single stem ginseng (G1) is the most common ginseng, but the phenomenon of multiple stems in ginseng (MG) is relatively rare. According to reports, external stimuli are the most common cause of MG, with chemical factors and mechanical trauma being the main external stimuli. After being stimulated by external factors, the latent buds on the rhizome of ginseng begin to sprout, resulting in the production of two or more rhizomes (Liu and Du, 1986). MG usually appears in ginseng aged 3 and 4, with 2 rhizomes being the most common (Zhang et al., 2012). Thanks to more ginseng leaves, MG has a higher photosynthetic rate and a significantly higher dry matter accumulation in roots than G1. Similarly, the content of ginsenosides in the roots of MG is also significantly higher than G1, indicating that MG has higher medicinal and economic value than G1 (Zhang et al., 2012).

Endogenous hormones are small molecule compounds in plants that regulate almost all life activities such as dormancy, reproduction, and secondary metabolite synthesis (Abbai et al., 2019). Correspondingly, plants can also cope with varying degrees of external environmental stimuli by regulating the accumulation of hormones such as salicylic acid and abscisic acid, and promoting the synthesis of secondary metabolites to resist environmental stress (Isah, 2019). In ginseng, Zhao et al. (2020) reported significant differential expression of important genes *90B*/*724B* in the brassinosteroid biosynthesis pathway between single stem and double stem ginseng. Compared with G1, Zou (2019) reported a significant upregulation of transcription factor *BZR1* in the brassinosteroid signaling pathway of double stem ginseng, indicating that *BZR1* played a key role in the production of MG.

The third year of ginseng growth is a crucial year (Yin et al., 2022), and the growth rate of ginseng is the fastest in this year, mainly reflected in the growth rate of ginsenoside content, root weight, and the number of lateral roots, which are the fastest compared to other years (Cho et al., 2007; Farh et al., 2018). At the same time, the incidence of ginseng diseases is also the highest in the third year of growth (Bao et al., 2020; Bian et al., 2022). Therefore, the third year of ginseng growth may be the year with the most frequent exchange of substances between roots and soil. At present, there are few reports on the factors influencing the appearance and quality of MG, previous reports had mainly focused on the spatiotemporal specific distribution of endogenous hormones (auxin, cytokinin, gibberellins, abscisic acid, and unicyclic lactone) (Zhao et al., 2020), seasonal changes in photosynthesis between G1 and MG (Li, 2012), comparison of carbohydrate and ginsenoside content in roots between G1 and MG (Liu and Du, 1986), and seasonal dynamics of antioxidant enzymes (superoxide dismutase, catalase, and peroxidase) between G1 and MG (Li, 2012). To our knowledge, there have been no studies reporting the differences in rhizosphere soil properties between G1 and MG and their potential impact on ginsenoside accumulation. In this study, we collected 3-year-old single stem, double stem, and triple stem ginseng and measured the content of ginsenosides in different parts. We also obtained rhizosphere and non-rhizosphere soils and measured soil abiotic factors and enzyme activity. We analyzed the correlation between soil factors and ginsenosides, and hypothesized that the interaction between ginseng and different soil microenvironments was the main factor leading to differences in ginsenoside content. At the same time, we also measured the endogenous hormone content in ginseng rhizomes to explore the relationship between endogenous hormones and soil environment. The results helped clarify the development value of MG and better guided agricultural production of MG.

## 2 Materials and methods

### 2.1 Overview of the experimental site

The experimental site is located in Zhujiabao, Qingyishan Town, Kuandian Manchu Autonomous County, Dandong City, Liaoning Province, China (124.63’E, 40.75’N), with an altitude of 305.12m (**Supplementary Figure S1A**). It belongs to the temperate continental monsoon climate, with distinct four seasons, warm winters and cool summers, and abundant sunshine as its main characteristics. The annual average temperature is 6.5°C, the annual effective accumulated temperature is 3000°C, the annual average precipitation is 1100 millimeters, mostly concentrated from June to August, the annual average humidity is 70%, and the average frost free period is 140 days per year (Shi, 2016).

### 2.2 Collection of ginseng and soil samples

The fields where ginseng was grown had previously been used for maize, and were then sown in the autumn using seeds of the “Damaya” cultivar with a row spacing of about 40cm between seeds. The field management measures for ginseng fields followed the standard method (GB/T 34789-2017). Specifically, the annual application of N, P, and K fertilizers to the soil from the second year of sowing, without supplementation of the remaining nutrients. The fertilizer was applied after ginseng leaf exhibition (early June), and the nitrogen fertilizer was added in the form of urea with a fertilizer rate of 23.74 g/m^2^; the phosphorus fertilizer was added in the form of superphosphate with a fertilizer rate of 68.02 g/m^2^; the potassium fertilizer was added in the form of potassium sulfate, the amount of fertilizer was 41.27 g/m^2^. No fertilization treatment was carried out, and irrigation was carried out every morning using tap water in the form of sprinkler irrigation to keep the bed moist but without water accumulation. From the leaf expansion period to the flowering period of ginseng, appropriate water supplementation was carried out after sunset in the evening according to its actual water utilization. Furthermore, during the growth period of ginseng, “carbendazim (systematic broad spectrum fungicide)” was applied, and from June to August, the focus was on the prevention and control of black spot disease, epidemic disease, and gray mold disease. Corresponding prevention and control agents were selected according to the incidence of ginseng disease, which were presented in GB/T 34789-2017. If ginseng was not infected, no additional agents were applied.

Ginseng and soil samples were collected at the end of the red fruit growth period of ginseng (September 1, 2024). Specifically, a total of 6 sets of replicates were designed, with 6 randomly ginseng fields, each with an area of approximately 60 m^2^ (2 m×30 m). 6–7 single stem ginsengs (G1, **Supplementary Figure S1B**), double stem ginsengs (G2, **Supplementary Figure S1B**), and three stem ginsengs (G3, **Supplementary Figure S1C**) were collected from each ginseng field using the “S” sampling method as one set of replicates. Regarding the “S” sampling method, specifically, six sampling points were symmetrically selected in each ginseng bed according to the “S” shape, and ginseng and soil samples were collected at each sampling point. The principle of “S” sampling method is to use its winding path to actively capture and cover the environmental gradients that may exist in the field. By extracting widely distributed samples, systematic errors are minimized to obtain a mixed sample that can represent the true situation of the entire field (Bao, 2000). Took 3 ginseng roots separately from each repetition, washed the surface soil with clean water, cut off the rhizomes, and put them into a liquid nitrogen tank filled with liquid nitrogen to brought back to the laboratory. Ginseng without rhizomes was not used for the analysis of ginsenoside content.

The root-shaking method was used to collect rhizosphere soil from the surface of ginseng roots. The collection method of non-rhizosphere soil (G0) was as follows: in each ginseng field, according to the “S” sampling method, soil without ginseng within a radius of 50–100 cm (0–15 cm soil layer) was collected. After mixing, it was used as a set of replicates, and a total of 6 replicate samples were collected in 6 ginseng fields.

Separated ginseng and soil, labeled them separately, then put them into sterile self sealing bags, and brought them back to the laboratory in a refrigerator with ice packs. After washing the soil on the surface of ginseng with deionized water, separated each set of repeated roots, stems, and leaves and let them air dry naturally. For dried ginseng stems, leaves, and roots, they were ground separately in a grinder and passed through a 0.18mm sieve for analysis of ginsenoside content. After homogenizing the soil, a portion was naturally air dried, then ground and analyzed through the corresponding sieve according to the requirements of the soil property determination regulations; the other part was stored in a refrigerator at 4 °C for future use.

### 2.3 Determination of soil physicochemical properties

For soil physical properties, the ring knife method was used to determine soil bulk density (BD) and field moisture capacity (FWC), while the drying method was used to determine mass moisture content (MWC). According to the reference standard method (LY/T1225-1999), the classification of soil aggregates (AGG) was determined using manual dry screening method. The specific method was as follows: sieve holes with diameters of 3.0mm, 2.00mm, 1.00mm, 0.85mm, 0.5mm, and 0.25mm from top to bottom, placed them on the bottom of the sieve, weighed about 100g of air dried soil and placed it on the 3mm sieve, and covered it with a manual sieve to divide the soil into 7 particle size groups, namely AGG > 3mm, 2–3 mm, 1–2 mm, 1-0.85mm, 0.85-0.5mm, and 0-0.25mm. The screened aggregates of each particle size were accurately weighed and the mass percentage of each particle size was calculated.

For soil chemical properties, pH and electrical conductivity (EC) were measured using a pH meter and conductivity meter under a soil to water ratio of 1:5. The alkaline hydrolysis diffusion method was used to determine the alkaline hydrolysis nitrogen (A-N), and the 1 mol/L ammonium acetate extraction-flame photometry method was used to determine the available potassium (A-K). The determination of phosphorus components referred to the grading method of Chang and Jackson (1957) and Olsen (1954). Specifically, the molybdenum antimony colorimetric method was used to determine the available phosphorus in soil. The leaching agents used for calcium bound phosphorus (Ca-P), iron bound phosphorus (Fe-P), aluminum bound phosphorus (Al-P), occluded phosphorus (O-P), available phosphorus (A-P), and inorganic phosphorus (I-P) were 0.5 mol/L H_2_SO_4_ solution, 0.1 mol/L NaOH solution, 0.5 mol/L ammonium fluoride solution, three acid mixtures (H_2_SO_4_: HClO: HNO_3_=1:2:7) digestion, 0.5 mol/L NaHCO_3_ solution, and 1 mol/L HCl solution, respectively. Organic carbon (SOC) was determined using an elemental analyzer (Vario EL III, Elementar, Hanau, Germany), while easily oxidizable organic carbon (EOC) was determined using a 333 mmol/L potassium permanganate solution oxidation method. The exchangeable total acid (E-TA), exchangeable hydrogen ion (E-H^+^), and exchangeable aluminum ion (E-Al^3+^) were determined by leaching with 1mol/L KCl and titration with 0.02 mol/L NaOH solution. Boiling water extraction-curcumin colorimetric method for determining available boron (A-B), calcium dihydrogen phosphate extraction-BaSO_4_ turbidimetric method for determining available sulfur (A-S), 0.025 mol/L citric acid solution extraction-silicon molybdenum blue colorimetric method for determining available silicon (A-Si), phenol disulfonic acid colorimetric method for determining nitrate nitrogen (NO_3_^–^N), KCl extraction-indophenol blue colorimetric method for determining ammonium nitrogen (NH_4_^+^-N), sodium pyrophosphate extraction-phenanthroline colorimetric method for determining complexed iron (C-Fe), pure water extraction-titration method for determining calcium ions (Ca^2+^), magnesium ions (Mg^2+^), chloride ions (Cl^-^), sulfate ions (SO_4_^2-^) and bicarbonate ion (HCO_3_^-^). The determination of cation exchange capacity (CEC) was referred to the “National Environmental Protection Standards of the People’s Republic of China” (HJ 889-2017). The reducing sugar content in soil was determined using the 3,5-dinitrosalicylic acid colorimetric method (Gusakov et al., 2011), the total sugar content was determined using the phenol-concentrated sulfuric acid method (Chow and Landhäusser, 2004), and the protein content was determined using the Bradford method. The specific measurement methods were presented in Bao (2000) and Lu (2000).

In order to better clarify the potential nutrient supply capacity of soil in G1 and MG, the content of total elements in the soil was measured. The content of total boron (T-B) was determined according to GB/T 3653.1-2024, the content of total manganese (T-Mn), total titanium (T-Ti), total calcium (T-Ca), total magnesium (T-Mg), total iron (T-Fe), total aluminum (T-Al), and total silicon (T-Si) was determined according to HJ 974-2018. The content of total zinc (T-Zn) was determined according to HJ 491-2019, and the determination of total europium (T-Eu), total lanthanum (T-La), total cerium (T-Ce), total praseodymium (T-Pr), total neodymium (T-Nd), total samarium (T-Sm), total gadolinium (T-Gd), total terbium (T-Tb), total dysprosium (T-Dy), total holmium (T-Ho), total erbium (T-Er), total thulium (T-Tm), total ytterbium (T-Yb), total lutetium (T-Lu), and total yttrium (T-Y) content referred to GB/T 18115.6-2023.

### 2.4 Determination of soil enzyme activity

The activities of some soil enzymes, including dehydrogenase (DHA, Cat: BC0390), phytase (Phy, Cat: BC5370), arylsulfatase (ASF, Cat: BC3995), uricase (UR, Cat: BC4410), polyphenol oxidase (PPO, Cat: BC0110), peroxidase (POD, Cat: BC0890), ascorbate peroxidase (APX, Cat: BC0220), lacase (LAC, Cat: BC1960), phenylalanine ammonia lyase (PAL, Cat: BC0210), fluorescein diacetate hydrolase (FDA, Cat: BC0485), hydroxylamine reductase (HR, Cat: BC3010), β - glucosidase (β - Glu, Cat: BC0160), superoxide dismutase (SOD, Cat: BC5160), glutaminase (GLS, Cat: BC3970), asparaginase (ASP, Cat: BC1600), were determined using the kit of Beijing Solabao Technology Co., Ltd.

The activity of amylase (AMY), α-amylase (α-AMY), β-amylase (β-AMY), cellulase (Cel), and invertase (Inv) was determined using the 3,5-dinitrosalicylic acid colorimetric method. The activity of acid phosphatase (ACP) was determined using the sodium phenylphosphate colorimetric method, the activity of nitrate reductase (NR) was determined using the phenol disulfonic acid colorimetric method, the activity of catalase (CAT) was determined using the potassium permanganate titration method, and the activity of ribonuclease (NUC) was determined using GB/T 34222-2017. The activity of urease (Ure) was determined using the phenol sodium hypochlorite colorimetric method, and the activity of protease (Prot) was determined using the ninhydrin colorimetric method. The specific measurement methods were presented in Guan et al. (1986).

### 2.5 Determination of ginsenoside content

According to the method for determining ginsenosides determined by the research group in the early stage (Xu et al., 2025), the ginsenoside content of roots, stems, and leaves of G1, G2, and G3 was measured. Using Thermo Ultimate 3000 high-performance liquid chromatography, the content of 11 types of ginsenosides was determined: Rg1, Re, Ro, Rb1, Rb2, Rb3, Rf, Rc, Rd, Rg3 (R-type), and Rh2 (R-type). The chromatographic column was Elite Hypersil ODS2 (250mm × 4.6mm, 5 μm). The composition of the mobile phase, gradient elution program, column temperature, injection volume, flow rate, and detection wavelength of the VWD detector were presented in **Supplementary Table S1**. The determination of total ginsenosides (TG) in ginseng was carried out according to the reference standard method (GB/T 18765-2015).

### 2.6 Determination of endogenous hormones in ginseng

Took the rhizomes from “2.2.” in a liquid nitrogen tank, ground them into powder using liquid nitrogen, and referred to the method described in the “National Drug Standard Draft for Determination of Plant Growth Regulator Residues (China)”. Used an Agilent 1290–6470 LC-MS/MS triple quadrupole liquid chromatography-mass spectrometry to determine the contents of anti-zeaxanthin, 6-benzylaminopurine, brassinolide, jasmonic acid, abscisic acid, gibberellin, 3-indoleacetic acid, and salicylic acid. The chromatographic column was Agilent ZORBAX Eclipse Plus C18 (2.1 × 100mm, 1.8 μm). The composition of the mobile phase, gradient elution program, column temperature, injection volume, flow rate, and mass spectrometry conditions were presented in **Supplementary Table S1**.

### 2.7 Data processing and statistical analysis

Measured all indicators three times and took the average as the final result. Used Microsoft Excel 2020 to organize the data, and then analyzed the data using SPSS 26.0 software. Firstly, performed Shapiro-Wilk normality test. Performed one-way analysis of variance (ANOVA) and Duncan’s multiple range test on data that conforms to normal distribution. Performed Dunnett’s T3 test on data that did not conform to normal distribution, with *p*<0.05 as the criterion for significant differences. The correlation analysis based on Spearman and Pearson test was also conducted in SPSS 26.0 software, and the difference was considered statistically significant when *p*<0.05. Performed random forest analysis using R (version 4.1.3) and the “randomForest” package (versions 4.7-1.1), and visualized it in the ggplot2 package (version 3.4.0). Performed principal component analysis (PCA) using the vegan package (v3.6.1) in R (v4.1.3), and visualized in the ggplot2 package (v3.3.3). The drawing materials were obtained in Cleanpng (https://www.cleanpng.com/). The visualization of data and analysis results was performed in GraphPad Prism 9.5.0 and SigmaPlot 10.0.

## 3 Results

### 3.1 Soil physicochemical properties

**Figure 1:**
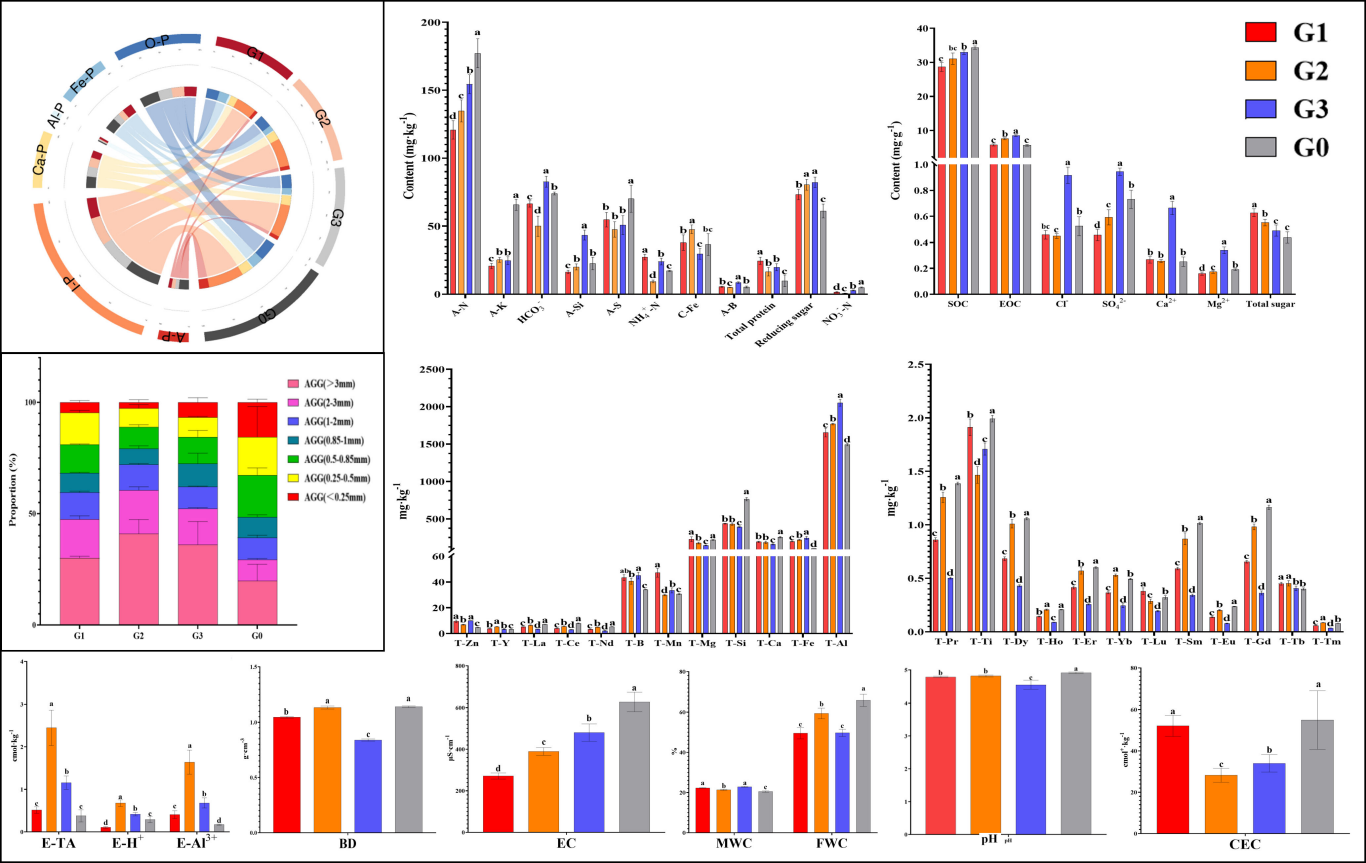
Physicochemical properties of different soil samples. Different lowercase letters above the same indicator bar chart indicated significant differences (*p*<0.05). The results indicate mean ± standard deviation (SD). BD, bulk density; FWC, field moisture capacity; MWC, mass moisture content; AGG, soil aggregates; EC, electrical conductivity; A-N, alkaline hydrolysis nitrogen; A-K, available potassium; Ca-P, calcium bound phosphorus; Fe-P, iron bound phosphorus; Al-P, aluminum bound phosphorus; O-P, occluded phosphorus; A-P, available phosphorus; I-P, inorganic phosphorus; SOC, Organic carbon; EOC, easily oxidizable organic carbon; E-TA, exchangeable total acid; E-H^+^, exchangeable hydrogen ion; E-Al^3+^, exchangeable aluminum ion; A-B, available boron; A-S, available sulfur; A-Si, available silicon; NO_3_^—^N, nitrate nitrogen; NH_4_^+^-N, ammonium nitrogen; C-Fe, complexed iron; Ca^2+^, calcium ions; Mg^2+^, magnesium ions; Cl^-^, chloride ions; SO_4_^2-^, sulfate ions; HCO_3_^-^, bicarbonate ion; CEC, cation exchange capacity; T-B, total boron; T-Mn, total manganese; T-Ti, total titanium; T-Ca, total calcium; T-Mg, total magnesium; T-Fe, total iron; T-Al, total aluminum; T-Si, total silicon; T-Zn, total zinc; T-Eu, total europium; T-La, total lanthanum; T-Ce, total cerium; T-Pr, total praseodymium; T-Nd, total neodymium; T-Sm, total samarium; T-Gd, total gadolinium; T-Tb, total terbium; T-Dy, total dysprosium; T-Ho, total holmium; T-Er, total erbium; T-Tm, total thulium; T-Yb, total ytterbium; T-Lu, total lutetium; T-Y, total yttrium. G0, non-rhizosphere soil; G1, rhizosphere soil of single stem ginseng; G2, rhizosphere soil of double stem ginseng; G3, rhizosphere soil of three stem ginseng.

The physicochemical properties of the soil were shown in **Figure 1**. In terms of conventional physicochemical properties, G0 (non-rhizosphere soil) had significantly higher levels of A-N, A-K, A-S, NO_3_^–^N, SOC, EC, FWC, pH, AGG (0.25-0.85mm), and phosphorus components (O-P, Fe-P, Ca-P, A-P, with significant differences not shown in the image) compared to rhizosphere soil. The characteristic of G1 was significantly higher content of NH_4_^+^-N, total protein, and total sugar. The content of C-Fe, AGG (>1mm), E-TA, E-H^+^, and E-Al^3+^ was significantly higher in G2 than in other groups. G3 had significantly higher levels of I-P, HCO_3_^-^, A-Si, A-B, EOC, Cl -, SO_4_^2-^, Ca^2+^, and Mg^2+^ content (*p*<0.05).

Among all elements, G0 had significantly higher levels of T-Si, T-Ca, T-Pr, T-Ti, T-Dy, T-Er, T-Sm, and T-Gd content. T-La, T-Ce, T-Nd, T-Fe, and T-Al were significantly the most abundant in G3. The content of T-Y and T-Tm was significantly highest in G2, while only the content of T-Mn in G1 was significantly higher than other groups (*p*<0.05).

### 3.2 Soil enzyme activity

**Figure 2:**
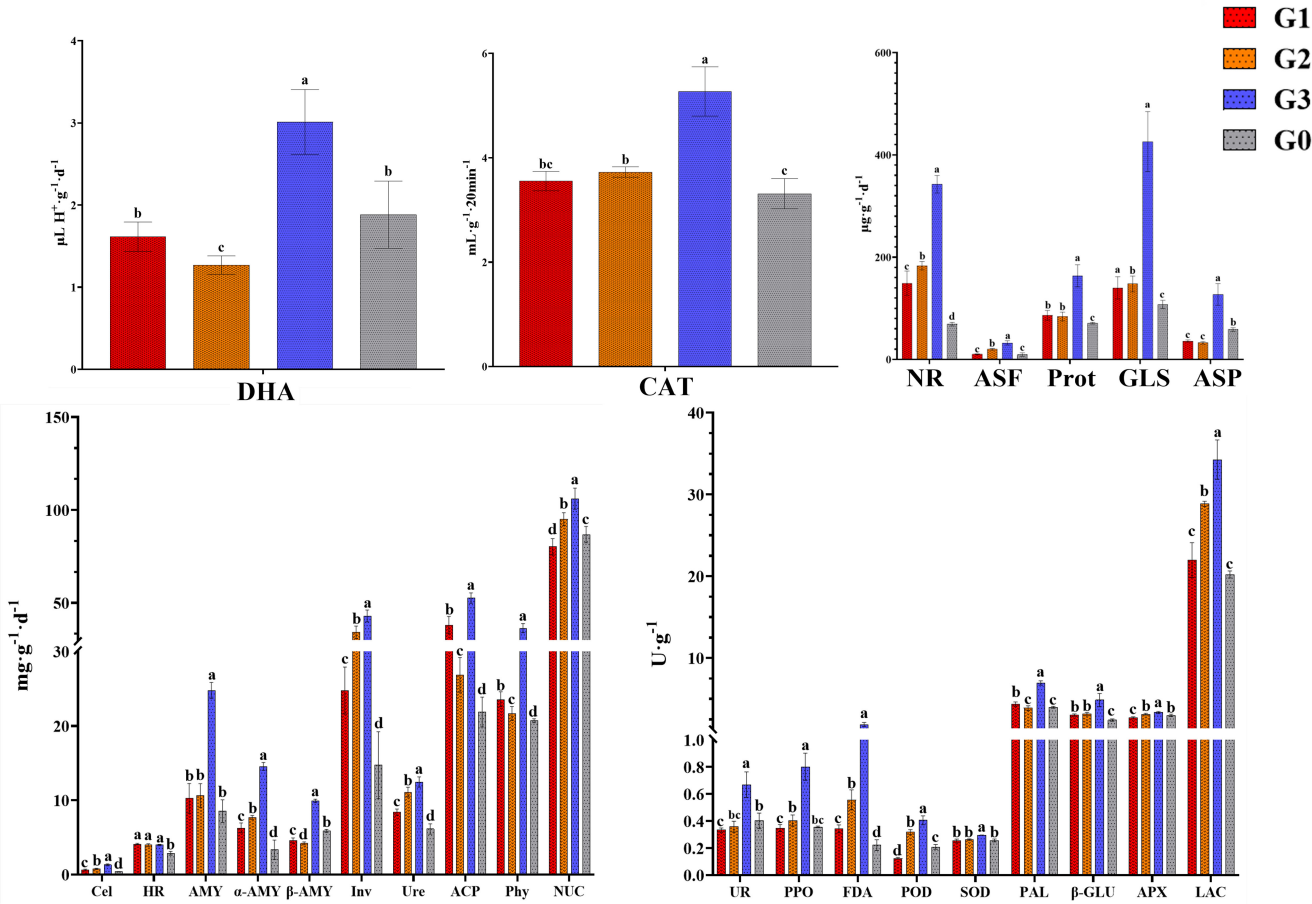
Enzyme activity of different soil samples. Different lowercase letters above the same indicator bar chart indicated significant differences (*p*<0.05). The results indicate mean ± SD. DHA, dehydrogenase; Phy, phytase; ASF, arylsulfatase; UR, uricase; PPO, polyphenol oxidase; POD, peroxidase; APX, ascorbate peroxidase; LAC, lacase; PAL, phenylalanine ammonia lyase; FDA, fluorescein diacetate hydrolase; HR, hydroxylamine reductase; β - Glu, β – glucosidase; SOD, superoxide dismutase; GLS, glutaminase; ASP, asparaginase; AMY; amylase; α-AMY, α-amylase; β-AMY, β-amylase; Cel, cellulase; Inv, invertase; ACP, acid phosphatase; NR, nitrate reductase; CAT, catalase; NUC, ribonuclease; Ure, urease; Prot, protease. G0, non-rhizosphere soil; G1, rhizosphere soil of single stem ginseng; G2, rhizosphere soil of double stem ginseng; G3, rhizosphere soil of three stem ginseng.

The soil enzyme activity was shown in **Figure 2**. We found a special phenomenon that the activity of almost all enzymes in G3 was significantly higher than other treatment groups (*p*<0.05, except for HR), indicating that the soil in G3 had higher nutrient cycling and energy transfer rates. In addition to G3, the activities of some enzymes related to carbon- (Inv), nitrogen- (NR, Prot, Ure), phosphorus- cycle (ACP), microbial activity (FDA), and oxidase (CAT, LAC) in the ginseng planting group (G1, G2) were significantly higher than those in G0 (*p*<0.05).

### 3.3 Morphology and ginsenoside content of ginseng with different stem numbers

**Figure 3:**
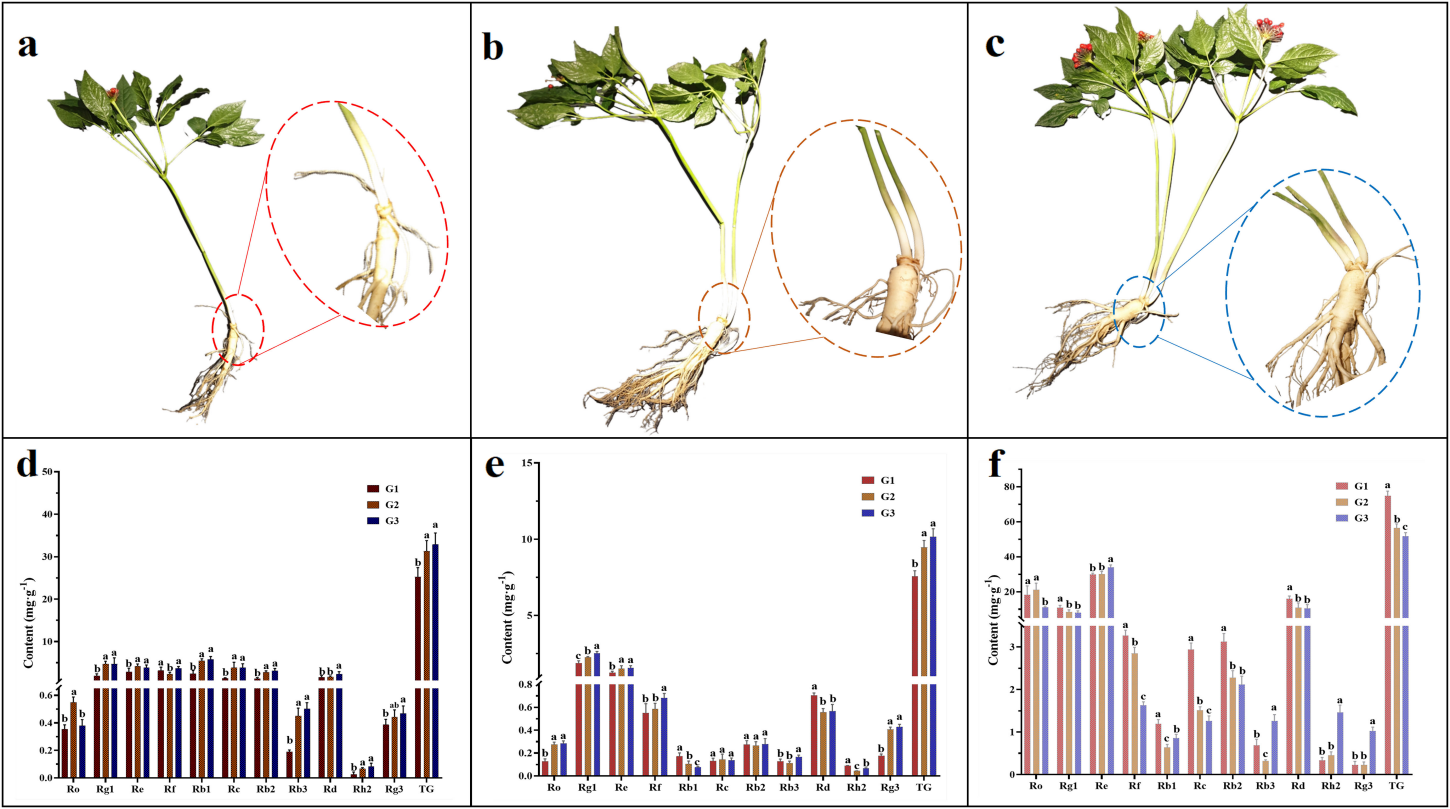
Appearance characteristics of ginseng with different stem numbers and the content of ginsenosides in different parts. **(a)** G1; **(b)** G2; **(c)** G3; The content of ginsenosides in roots **(d)**, stems **(e)**, and leaves **(f)**. Different lowercase letters above the same indicator bar chart indicated significant differences (*p<0.05*). The results indicate mean ± SD. G1, single stem ginseng; G2, double stem ginseng; G3, three stem ginseng.

There were significant differences in the morphology of the aboveground parts of G1, G2, and G3 (**Figures 3A-C**). The results of ginsenosides content in roots (**Figure 3D**) showed that most ginsenosides (Rg1, Re, Rb1, Rc, Rb2, Rb3, Rh2, TG) in MG was significantly higher than that in G1 (*p*<0.05). The content of TG in G2 and G3 reached 32.88 mg/g and 31.29 mg/g, which were 1.30 and 1.24 times that of G1, respectively. Additionally, it is worth noting that G2 had a significantly higher content of ginsenoside Ro, while G3 had a significantly higher content of ginsenosides Rd and Rg3 (*p*<0.05).

In the stem (**Figure 3E**), the content of ginsenosides Ro and Re in MG was significantly higher than that in G1. In addition, G3 exhibited significantly higher levels of ginsenosides Rg1, Rf, Rb3, Rg3, and TG, with TG content (10.17 mg/g) being 1.35 and 1.08 times higher than G1 (7.56 mg/g) and G2 (9.46 mg/g), respectively. Finally, the content of ginsenosides Rb1, Rd, and Rh2 was significantly higher in G1 (*p*<0.05).

The ginsenosides of ginseng with different stem numbers in the leaves were significantly different from those in the roots and stems (**Figure 3F**). The content of most ginsenosides (Rg1, Rf, Rb1, Rc, Rb2, Rd, and TG) in G1 was significantly higher than that in G2 and G3, while the content of ginsenosides Re, Rb3, Rh2, and Rg3 in G3 was significantly higher than that in G1 and G2. In terms of TG content, G1 (74.78 mg/g) was 1.32 and 1.45 times higher than G2 (56.49 mg/g) and G3 (51.66 mg/g).

### 3.4 Endogenous hormone content of ginseng with different stem numbers

The content of 8 endogenous hormones in the rhizomes was determined (**Table 1**), and the contents of anti-zeaxanthin, gibberellin, 6-benzylaminopurine, abscisic acid, and jasmonic acid were all below the minimum detection limit. Therefore, we mainly analyzed the content of salicylic acid, 3-indoleacetic acid, and brassinolide. The content of salicylic acid in G2 and G3 was significantly higher than that in G1, while the content of 3-indoleacetic acid showed no significant difference among the three groups (*p* > 0.05). For brassinolide, the content was G1>G3>G2, the difference was significant (*p* <0.05).

**Table 1:** Content of endogenous hormones in ginseng rhizomes.

Endogenous hormones	Unit	G1	G2	G3
Anti-zeaxanthin	μg·g^-1^	–	–	–
Gibberellin		–	–	–
Salicylic acid		0.125±0.007b	0.138±0.006a	0.142±0.005a
3-indoleacetic acid		0.023±0.001a	0.023±0.002a	0.021±0.002a
6-benzylaminopurine		–	–	–
Abscisic acid		–	–	–
Brassinolide		0.201±0.006a	0.143±0.007c	0.174±0.006b
Jasmonic acid		–	–	–

Different lowercase letters represented significant differences *(p<0.05*) in the same indicator, and “-” indicated that the result was below the minimum detection limit. The results indicate mean ± SD. G1, single stem ginseng; G2, double stem ginseng; G3, three stem ginseng.

### 3.5 Ternary plot analysis

**Figure 4:**
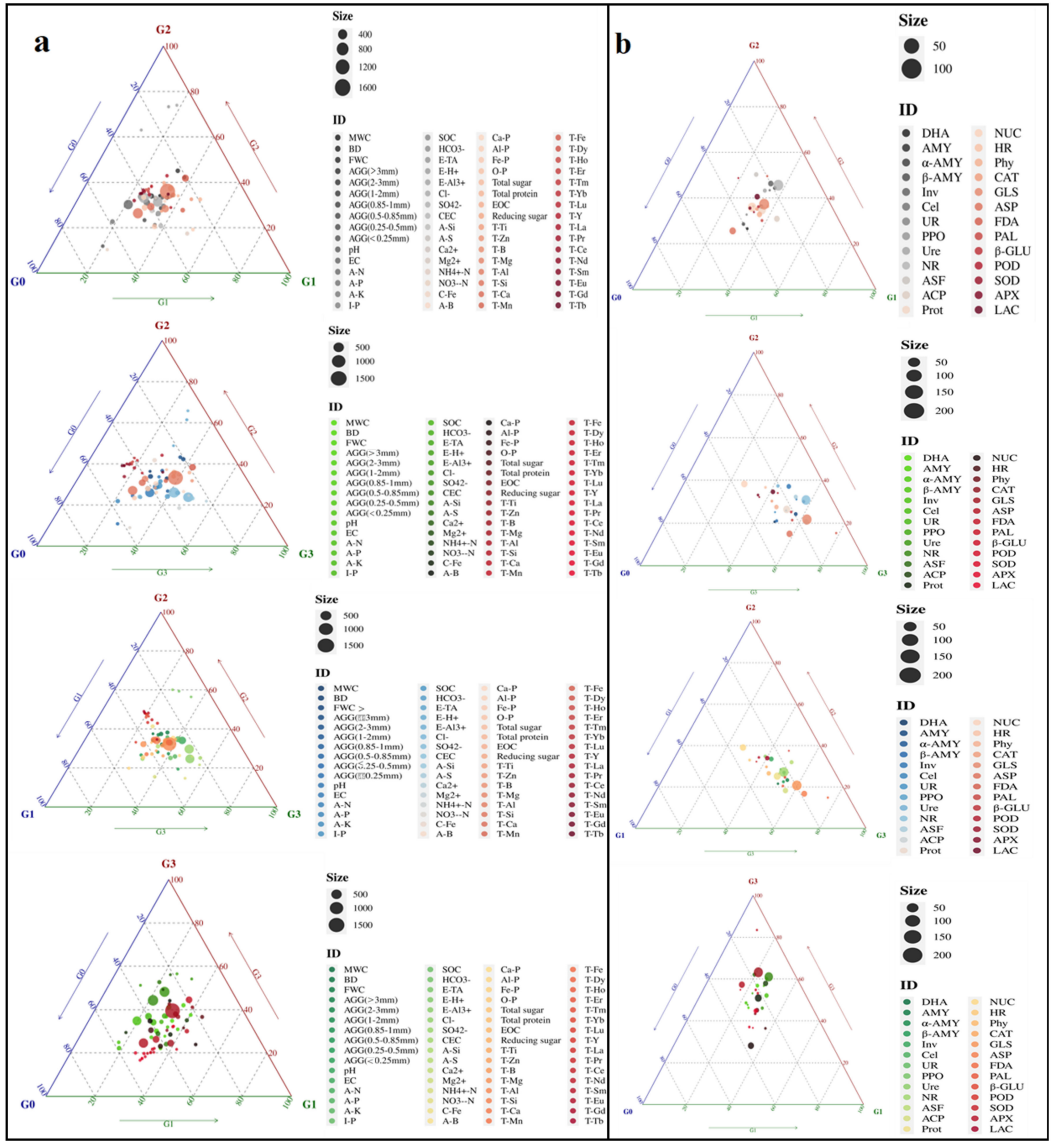
Ternary plot displaying the abundance of indicators for different soil and ginseng samples. **(a)** Soil physicochemical properties; **(b)** Soil enzyme activity; By observing the position of points in the graph, the relative content of each component can be determined. The closer a point is to a certain vertex, the higher the relative content of that component. The size of the midpoint of the triangle corresponds to the mean abundance of the elements in the three groups. BD, bulk density; FWC, field moisture capacity; MWC, mass moisture content; AGG, soil aggregates; EC, electrical conductivity; A-N, alkaline hydrolysis nitrogen; A-K, available potassium; Ca-P, calcium bound phosphorus; Fe-P, iron bound phosphorus; Al-P, aluminum bound phosphorus; O-P, occluded phosphorus; A-P, available phosphorus; I-P, inorganic phosphorus; SOC, Organic carbon; EOC, easily oxidizable organic carbon; E-TA, exchangeable total acid; E-H^+^, exchangeable hydrogen ion; E-Al^3+^, exchangeable aluminum ion; A-B, available boron; A-S, available sulfur; A-Si, available silicon; NO_3_^—^N, nitrate nitrogen; NH_4_^+^-N, ammonium nitrogen; C-Fe, complexed iron; Ca^2+^, calcium ions; Mg^2+^, magnesium ions; Cl^-^, chloride ions; SO_4_^2-^, sulfate ions; HCO_3_^-^, bicarbonate ion; CEC, cation exchange capacity; T-B, total boron; T-Mn, total manganese; T-Ti, total titanium; T-Ca, total calcium; T-Mg, total magnesium; T-Fe, total iron; T-Al, total aluminum; T-Si, total silicon; T-Zn, total zinc; T-Eu, total europium; T-La, total lanthanum; T-Ce, total cerium; T-Pr, total praseodymium; T-Nd, total neodymium; T-Sm, total samarium; T-Gd, total gadolinium; T-Tb, total terbium; T-Dy, total dysprosium; T-Ho, total holmium; T-Er, total erbium; T-Tm, total thulium; T-Yb, total ytterbium; T-Lu, total lutetium; T-Y, total yttrium. DHA, dehydrogenase; Phy, phytase; ASF, arylsulfatase; UR, uricase; PPO, polyphenol oxidase; POD, peroxidase; APX, ascorbate peroxidase; LAC, lacase; PAL, phenylalanine ammonia lyase; FDA, fluorescein diacetate hydrolase; HR, hydroxylamine reductase; β - Glu, β – glucosidase; SOD, superoxide dismutase; GLS, glutaminase; ASP, asparaginase; AMY; amylase; α-AMY, α-amylase; β-AMY, β-amylase; Cel, cellulase; Inv, invertase; ACP, acid phosphatase; NR, nitrate reductase; CAT, catalase; NUC, ribonuclease; Ure, urease; Prot, protease. G0, non-rhizosphere soil; G1, rhizosphere soil of single stem ginseng; G2, rhizosphere soil of double stem ginseng; G3, rhizosphere soil of three stem ginseng.

**Figure 5:**
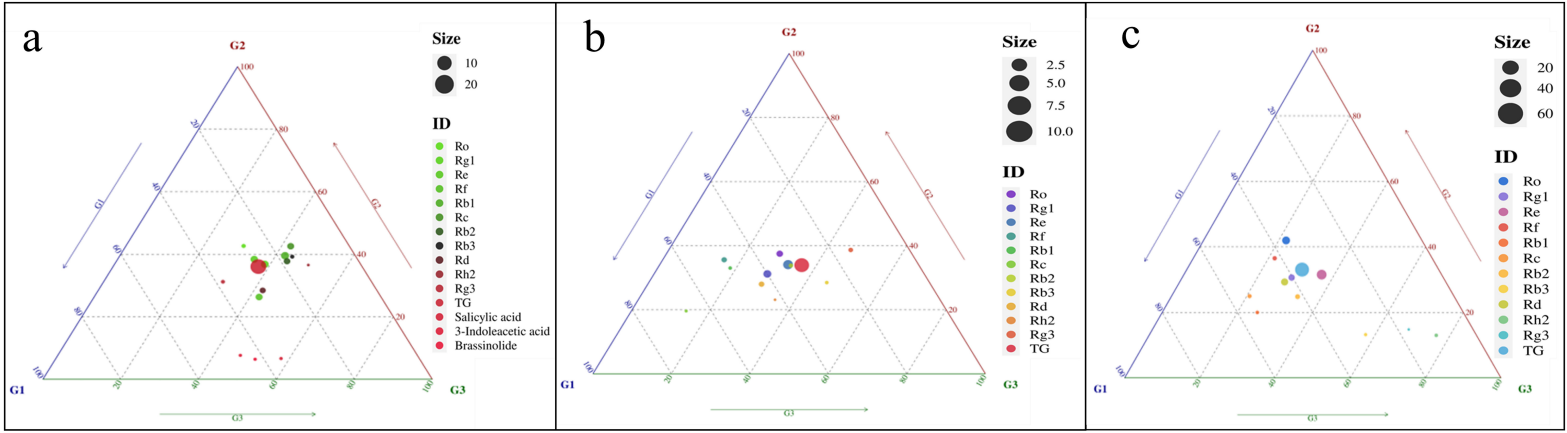
Ternary plot displaying the abundance of indicators for different ginseng samples. **(a)** Content of chemical components in roots; **(b)** Chemical composition content in stems; **(c)** Chemical composition content in leaves. G1, single stem ginseng; G2, double stem ginseng; G3, three stem ginseng.

In order to clarify the differences in soil properties and quality indicators of ginseng among different samples, a ternary plot was drawn (**Figure 4**). The results showed that the higher abundance of soil physicochemical properties was mainly concentrated in G0 (**Figure 4A**), while the higher soil enzyme activity was mainly concentrated in G3, followed by G1 and G2, suggesting that G3 had higher microbial activity to support nutrient cycling and energy flow (**Figure 4B**). The content of ginsenosides in roots was mainly enriched in G3; in rhizomes, high levels of endogenous hormones were mainly enriched in G3 (**Figure 4C**). In stems and leaves, the enrichment of ginsenoside content was greater in G1 and G2 (**Figures 5A-C**).

### 3.6 Principal component analysis

**Figure 6:**
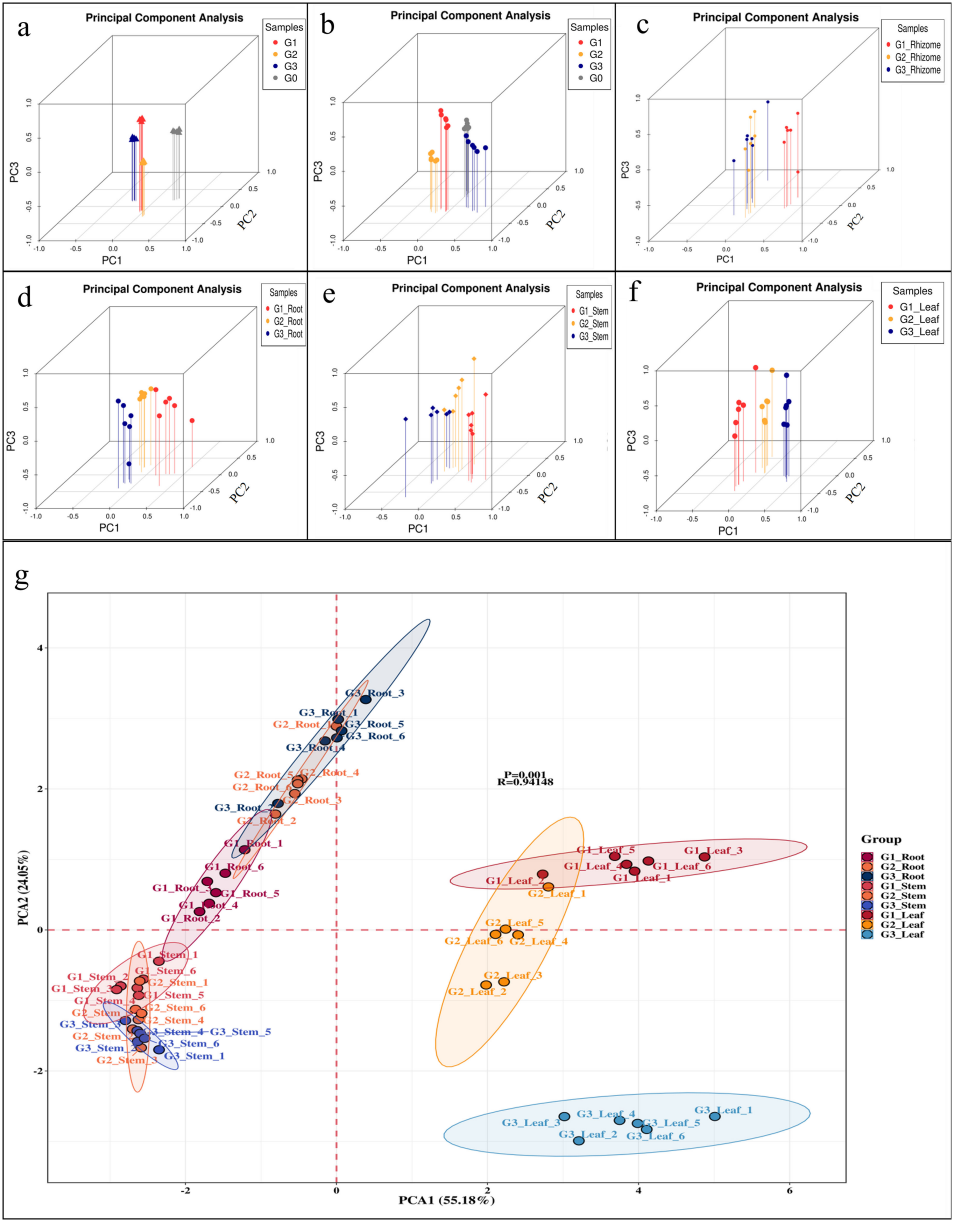
Principal component analysis (PCA) of rhizosphere and non-rhizosphere soil properties, endogenous hormones, and ginsenosides from different parts. **(a)** Soil physicochemical properties; **(b)** Soil enzyme activity; **(c)** Endogenous hormones; **(d-g)** The roots, stems, and leaves of ginseng. **(a-c)** G0, non-rhizosphere soil; G1, rhizosphere soil of single stem ginseng; G2, rhizosphere soil of double stem ginseng; G3, rhizosphere soil of three stem ginseng. **(d-g)** G1, single stem ginseng; G2, double stem ginseng; G3, three stem ginseng.

In order to clarify the differences in soil properties and ginseng quality, principal component analysis (PCA) was conducted. The PCA results of soil physicochemical properties (**Figure 6A**) showed significant differences among different groups. The PCA results of soil enzyme activity (**Figure 6B**) showed that although the differences between different groups could be roughly distinguished, the differences in enzyme activity between G3 and G0 were relatively small.

The PCA results of endogenous hormone content in rhizomes (**Figure 6C**) showed significant differences between G1 and MG (G2, G3), but there was overlap between G2 and G3. The PCA results of ginsenoside content in the roots (**Figure 6D**) showed that G3 was clearly distinguished from G1 and G2, and although there were significant differences between G1 and G2, their similarity was higher. The PCA results of ginsenoside content in stems (**Figure 6E**) were exactly opposite to those in roots, with significant differences between G1 and MG, and partial overlap between G2 and G3. The PCA results of ginsenoside content in leaves (**Figure 6F**) showed that although there were significant differences between different samples, the difference between G1 and G3 was even greater.

For the sake of clarifying the differences in the content of ginsenosides in three different parts, a comprehensive analysis was conducted (**Figure 6G**). The results were roughly the same as those analyzed separately (**Figures 6D-F**). In addition, the roots, stems, and leaves were each located in a quadrant, and the differences between leaves and roots and stems were significant.

### 3.7 Random forest analysis

**Figure 7:**
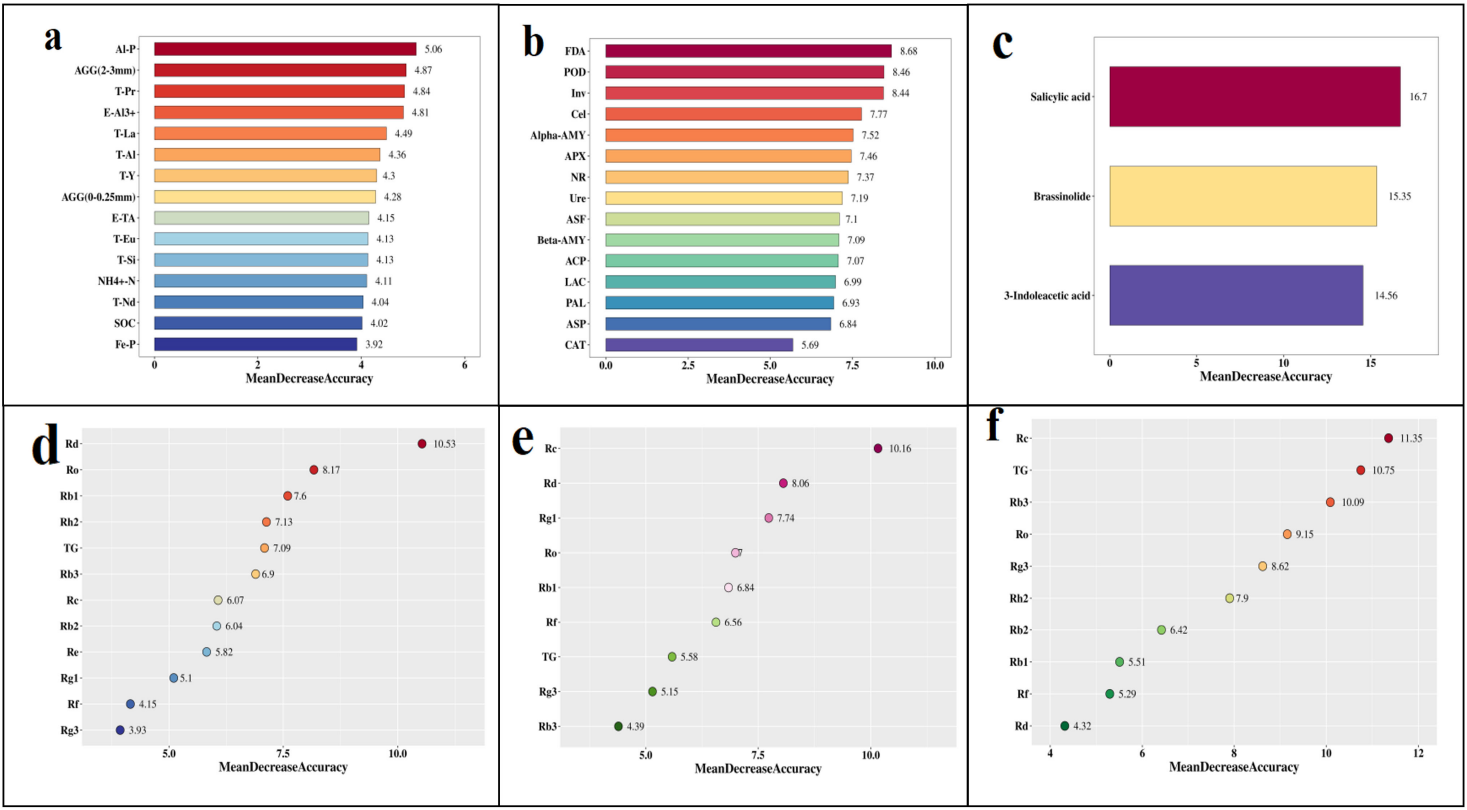
Random forest analysis of different parts of soil and ginseng. **(a)** Soil physicochemical properties; **(b)** Soil enzyme activity properties; Endogenous hormones in the rhizomes **(c)** and ginsenosides content in the roots **(d)**, stems **(e)**, and leaves **(f)** of ginseng. The MeanDeceaseAccuracy on the horizontal axis represents the relative importance of the indicator in classification, with a higher value indicating a greater contribution of the indicator to the inter group differences. **(a-b)** Al-P, aluminum bound phosphorus; AGG, soil aggregates; T-Pr, total praseodymium; E-Al^3+^, exchangeable aluminum ion; T-La, total lanthanum; T-Al, total aluminum; T-Y, total yttrium; E-TA, exchangeable total acid; T-Eu, total europium; T-Si, total silicon; NH_4_^+^-N, ammonium nitrogen; T-Nd, total neodymium; SOC, Organic carbon; Fe-P, iron bound phosphorus; FDA, fluorescein diacetate hydrolase; POD, peroxidase; Inv, invertase; Cel, cellulase; Alpha-AMY, α-amylase; APX, ascorbate peroxidase; NR, nitrate reductase; Ure, urease; ASF, arylsulfatase; Beta-AMY, β-amylase; ACP, acid phosphatase; LAC, lacase; PAL, phenylalanine ammonia lyase; ASP, asparaginase; CAT, catalase.

The results of PCA had confirmed significant differences among samples from different groups, but the characteristic factors that caused these differences were not yet clear. Here, we used random forest analysis to identify the characteristic factors of these differences (**Figure 7**). In soil physicochemical properties, Al-P, AGG (2–3 mm), T-Pr, E-Al^3+^, and T-La were characteristic factors of differences (**Figure 7A**). In soil enzyme activity, FDA, POD, and Inv were characteristic factors of differences (**Figure 7B**).

The main difference in endogenous hormone content in rhizomes was salicylic acid (**Figure 7C**), the characteristic factors for the difference in ginsenoside content in roots were ginsenosides Rd and Ro (**Figure 7D**), the characteristic factors for the difference in ginsenoside content in stems were ginsenosides Rc and Rd (**Figure 7E**), and the characteristic factors for the difference in ginsenoside content in leaves were ginsenosides Rc, TG, and Rb3 (**Figure 7F**).

### 3.8 Correlation analysis

#### 3.8.1 Correlation analysis between chemical components in roots and soil properties

**Figure 8:**
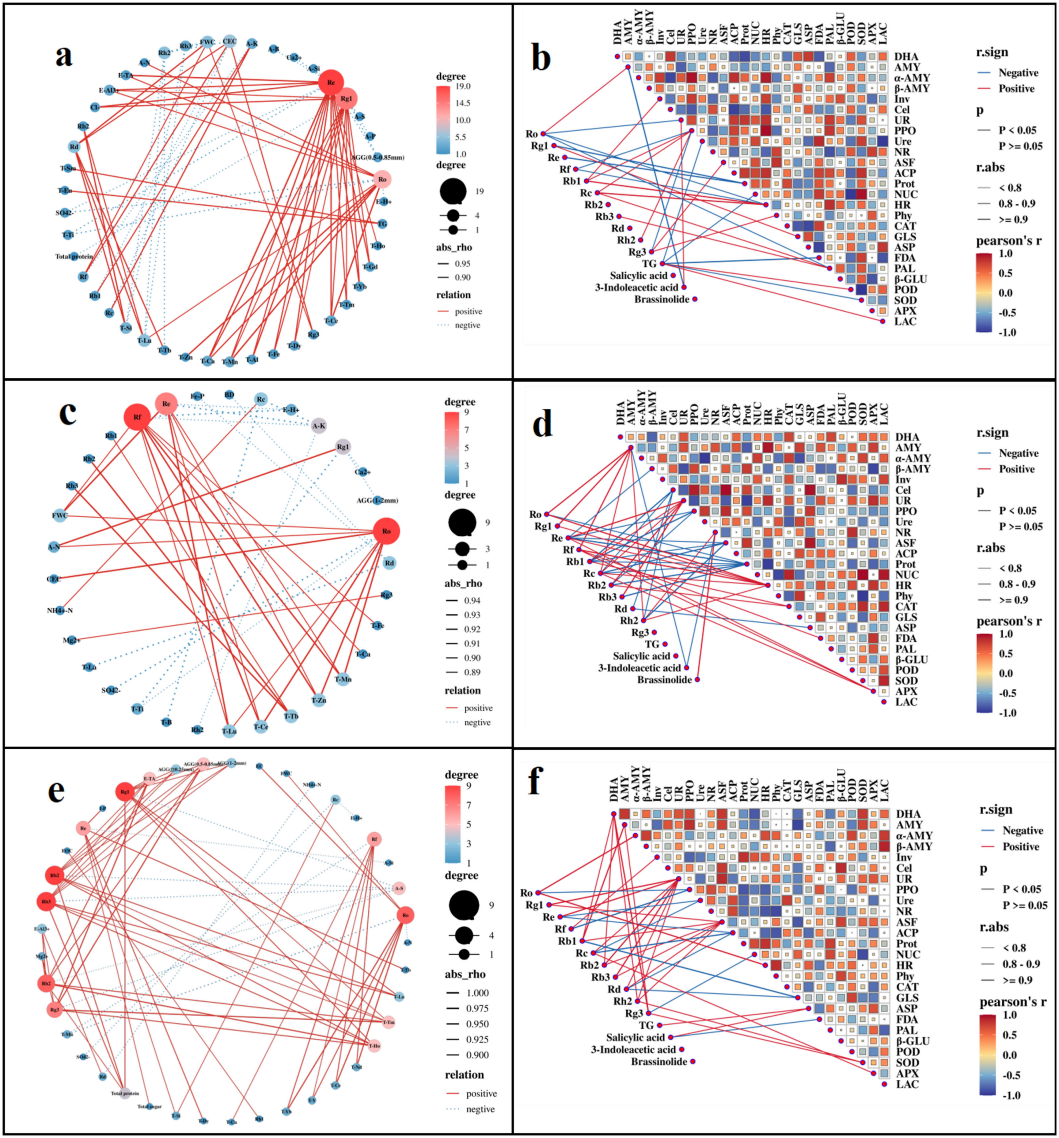
Correlation analysis of ginsenosides and endogenous hormones in roots with soil physicochemical properties (**a**: G1; **c**: G2; **e**: G3) and enzyme activity (**b**: G1; **d**: G2; **f**: G3). In the relationship between different indicators, the red line represents positive correlation and the blue line represents negative correlation, presenting only results with *p<0.05*. In the correlation network between ginsenosides and soil physicochemical properties **(a, c, e)**, the size of a node and the depth of its red color are positively correlated with the number of connections, the more connections there are, the larger the node and the darker the red color. BD, bulk density; FWC, field moisture capacity; MWC, mass moisture content; AGG, soil aggregates; EC, electrical conductivity; A-N, alkaline hydrolysis nitrogen; A-K, available potassium; Ca-P, calcium bound phosphorus; Fe-P, iron bound phosphorus; Al-P, aluminum bound phosphorus; O-P, occluded phosphorus; A-P, available phosphorus; I-P, inorganic phosphorus; SOC, Organic carbon; EOC, easily oxidizable organic carbon; E-TA, exchangeable total acid; E-H^+^, exchangeable hydrogen ion; E-Al^3+^, exchangeable aluminum ion; A-B, available boron; A-S, available sulfur; A-Si, available silicon; NO_3_^—^N, nitrate nitrogen; NH_4_^+^-N, ammonium nitrogen; C-Fe, complexed iron; Ca^2+^, calcium ions; Mg^2+^, magnesium ions; Cl^-^, chloride ions; SO_4_^2-^, sulfate ions; HCO_3_^-^, bicarbonate ion; CEC, cation exchange capacity; T-B, total boron; T-Mn, total manganese; T-Ti, total titanium; T-Ca, total calcium; T-Mg, total magnesium; T-Fe, total iron; T-Al, total aluminum; T-Si, total silicon; T-Zn, total zinc; T-Eu, total europium; T-La, total lanthanum; T-Ce, total cerium; T-Pr, total praseodymium; T-Nd, total neodymium; T-Sm, total samarium; T-Gd, total gadolinium; T-Tb, total terbium; T-Dy, total dysprosium; T-Ho, total holmium; T-Er, total erbium; T-Tm, total thulium; T-Yb, total ytterbium; T-Lu, total lutetium; T-Y, total yttrium; DHA, dehydrogenase; Phy, phytase; ASF, arylsulfatase; UR, uricase; PPO, polyphenol oxidase; POD, peroxidase; APX, ascorbate peroxidase; LAC, lacase; PAL, phenylalanine ammonia lyase; FDA, fluorescein diacetate hydrolase; HR, hydroxylamine reductase; β - Glu, β – glucosidase; SOD, superoxide dismutase; GLS, glutaminase; ASP, asparaginase; AMY; amylase; α-AMY, α-amylase; β-AMY, β-amylase; Cel, cellulase; Inv, invertase; ACP, acid phosphatase; NR, nitrate reductase; CAT, catalase; NUC, ribonuclease; Ure, urease; Prot, protease.

To clarify the potential impact of soil properties on the accumulation of ginsenosides, we conducted a correlation analysis based on Spearman test (**Figure 8**, **Supplementary Table S2**). In G1, ginsenosides Ro, Rg1, and Re were most significantly affected by soil abiotic factors (**Figure 8A**), while PPO, HR, and PAL were the most important soil enzymes affecting ginsenoside accumulation (**Figure 8B**). In G2, ginsenosides Ro, Re, and Rf were most significantly affected by soil abiotic factors (**Figure 8C**), while AMY, Cel, and Prot were the most important soil enzymes affecting ginsenoside accumulation (**Figure 8D**). In G3, ginsenosides Rg1, Rb2, and Rb3 were most significantly affected by soil abiotic factors (**Figure 8E**), while ASF, UR, and DHA were the most important soil enzymes affecting ginsenoside accumulation (**Figure 8F**).

It is worth noting that the endogenous hormone content in rhizomes seemed to be minimally affected by soil abiotic factors, as their correlation with abiotic factors had not reached a significant level.

#### 3.8.2 Correlation analysis between ginsenosides in stems and soil properties

**Figure 9:**
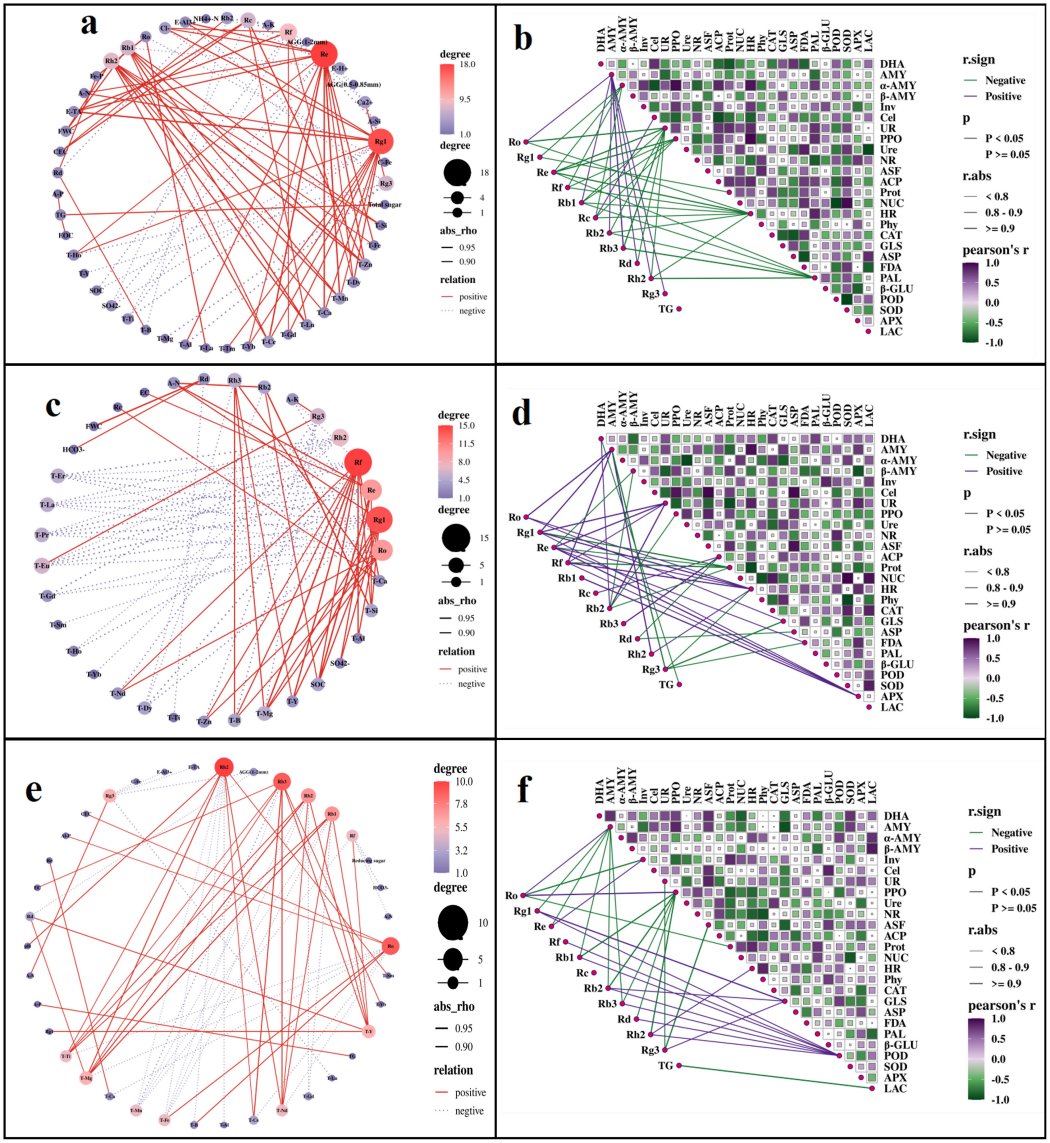
Correlation analysis of ginsenosides in stems with soil physicochemical properties (**a**: G1; **c**: G2; **e**: G3) and enzyme activity (**b**: G1; **d**: G2; **f**: G3). In the correlation network between ginsenosides and soil physicochemical properties **(a, c, e)**, the red solid line represents positive correlation, and the purple dashed line represents negative correlation, the size of a node and the depth of its red color are positively correlated with the number of connections, the more connections there are, the larger the node and the darker the red color. In the correlation analysis between ginsenosides and soil enzyme activity, the purple line represents the correlation, and the green line represents the negative correlation. When the p-value of the correlation between two indicators is greater than 0.05, the line connecting them is not displayed. BD, bulk density; FWC, field moisture capacity; MWC, mass moisture content; AGG, soil aggregates; EC, electrical conductivity; A-N, alkaline hydrolysis nitrogen; A-K, available potassium; Ca-P, calcium bound phosphorus; Fe-P, iron bound phosphorus; Al-P, aluminum bound phosphorus; O-P, occluded phosphorus; A-P, available phosphorus; I-P, inorganic phosphorus; SOC, Organic carbon; EOC, easily oxidizable organic carbon; E-TA, exchangeable total acid; E-H^+^, exchangeable hydrogen ion; E-Al^3+^, exchangeable aluminum ion; A-B, available boron; A-S, available sulfur; A-Si, available silicon; NO_3_^—^N, nitrate nitrogen; NH_4_^+^-N, ammonium nitrogen; C-Fe, complexed iron; Ca^2+^, calcium ions; Mg^2+^, magnesium ions; Cl^-^, chloride ions; SO_4_^2-^, sulfate ions; HCO_3_^-^, bicarbonate ion; CEC, cation exchange capacity; T-B, total boron; T-Mn, total manganese; T-Ti, total titanium; T-Ca, total calcium; T-Mg, total magnesium; T-Fe, total iron; T-Al, total aluminum; T-Si, total silicon; T-Zn, total zinc; T-Eu, total europium; T-La, total lanthanum; T-Ce, total cerium; T-Pr, total praseodymium; T-Nd, total neodymium; T-Sm, total samarium; T-Gd, total gadolinium; T-Tb, total terbium; T-Dy, total dysprosium; T-Ho, total holmium; T-Er, total erbium; T-Tm, total thulium; T-Yb, total ytterbium; T-Lu, total lutetium; T-Y, total yttrium; DHA, dehydrogenase; Phy, phytase; ASF, arylsulfatase; UR, uricase; PPO, polyphenol oxidase; POD, peroxidase; APX, ascorbate peroxidase; LAC, lacase; PAL, phenylalanine ammonia lyase; FDA, fluorescein diacetate hydrolase; HR, hydroxylamine reductase; β - Glu, β – glucosidase; SOD, superoxide dismutase; GLS, glutaminase; ASP, asparaginase; AMY; amylase; α-AMY, α-amylase; β-AMY, β-amylase; Cel, cellulase; Inv, invertase; ACP, acid phosphatase; NR, nitrate reductase; CAT, catalase; NUC, ribonuclease; Ure, urease; Prot, protease.

**Figure 9** and **Supplementary Table S3** presented the correlation analysis results between ginsenosides in stems and soil properties. In G1, ginsenosides Rg1 and Re were most significantly affected by soil abiotic factors (**Figure 9A**), while AMY, HR, and PAL were the most noteworthy soil enzymes affecting ginsenoside accumulation (**Figure 9B**). In G2, ginsenosides Ro, Re, Rf, and Rg1 were most significantly affected by soil abiotic factors (**Figure 9C**), while AMY, APX, HR, and Prot were the most noteworthy soil enzymes affecting ginsenoside accumulation (**Figure 9D**). In G3, ginsenosides Rh2, Rb3, and Ro were most significantly affected by soil abiotic factors (**Figure 9E**), while AMY, PPO, and POD were the most noteworthy soil enzymes affecting ginsenoside accumulation (**Figure 9F**).

#### 3.8.3 Correlation analysis between ginsenosides in leaves and soil properties

**Figure 10:**
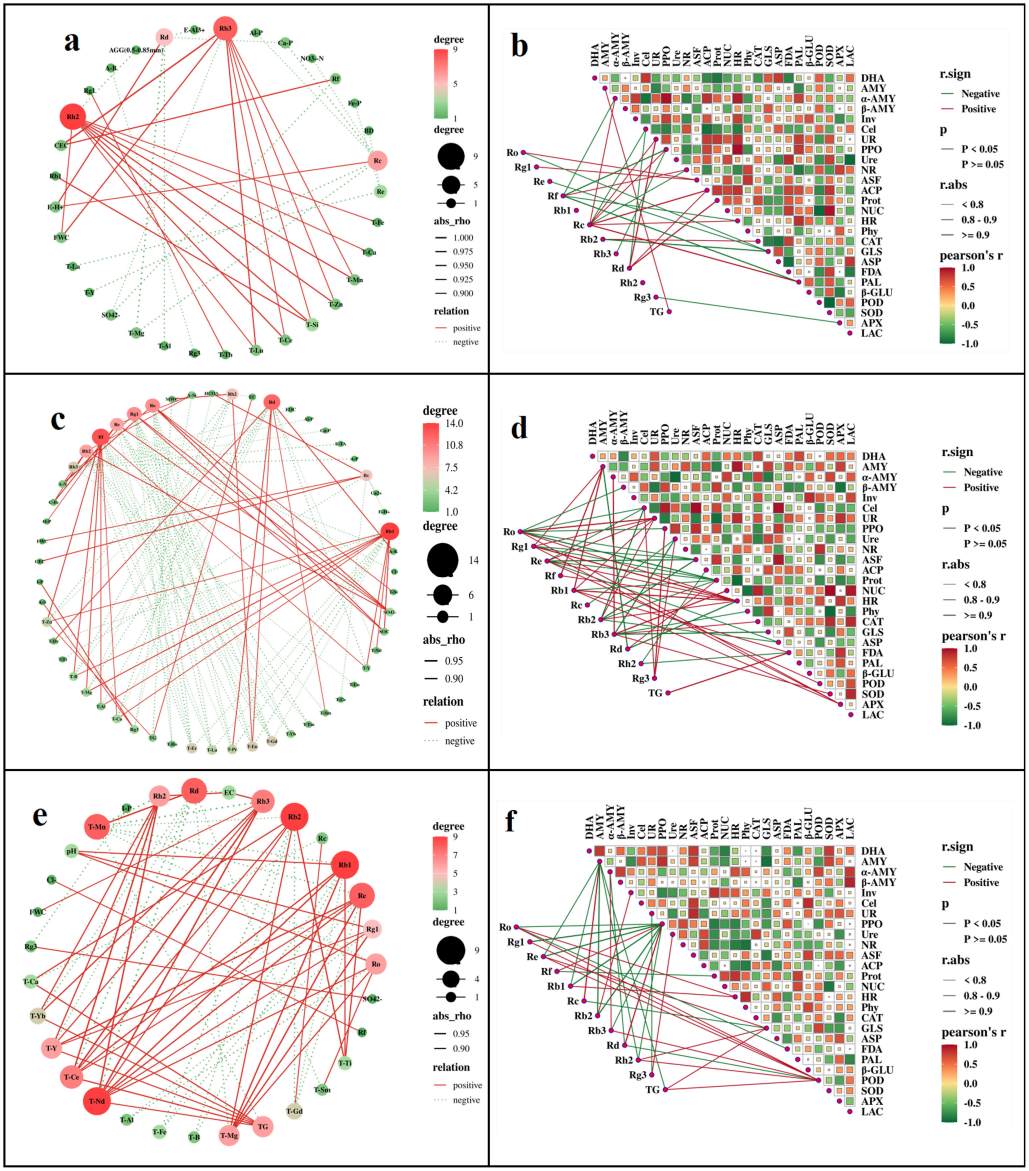
Correlation analysis of ginsenosides in leaves with soil physicochemical properties (**a**: G1; **c**: G2; **e**: G3) and enzyme activity (**b**: G1; **d**: G2; **f**: G3). In the relationship between different indicators, the red line represents positive correlation and the green line represents negative correlation, presenting only results with p<0.05. In the correlation network between ginsenosides and soil physicochemical properties **(a, c, e)**, the size of a node and the depth of its red color are positively correlated with the number of connections, the more connections there are, the larger the node and the darker the red color. BD, bulk density; FWC, field moisture capacity; MWC, mass moisture content; AGG, soil aggregates; EC, electrical conductivity; A-N, alkaline hydrolysis nitrogen; A-K, available potassium; Ca-P, calcium bound phosphorus; Fe-P, iron bound phosphorus; Al-P, aluminum bound phosphorus; O-P, occluded phosphorus; A-P, available phosphorus; I-P, inorganic phosphorus; SOC, Organic carbon; EOC, easily oxidizable organic carbon; E-TA, exchangeable total acid; E-H^+^, exchangeable hydrogen ion; E-Al^3+^, exchangeable aluminum ion; A-B, available boron; A-S, available sulfur; A-Si, available silicon; NO_3_^—^N, nitrate nitrogen; NH_4_^+^-N, ammonium nitrogen; C-Fe, complexed iron; Ca^2+^, calcium ions; Mg^2+^, magnesium ions; Cl^-^, chloride ions; SO_4_^2-^, sulfate ions; HCO_3_^-^, bicarbonate ion; CEC, cation exchange capacity; T-B, total boron; T-Mn, total manganese; T-Ti, total titanium; T-Ca, total calcium; T-Mg, total magnesium; T-Fe, total iron; T-Al, total aluminum; T-Si, total silicon; T-Zn, total zinc; T-Eu, total europium; T-La, total lanthanum; T-Ce, total cerium; T-Pr, total praseodymium; T-Nd, total neodymium; T-Sm, total samarium; T-Gd, total gadolinium; T-Tb, total terbium; T-Dy, total dysprosium; T-Ho, total holmium; T-Er, total erbium; T-Tm, total thulium; T-Yb, total ytterbium; T-Lu, total lutetium; T-Y, total yttrium; DHA, dehydrogenase; Phy, phytase; ASF, arylsulfatase; UR, uricase; PPO, polyphenol oxidase; POD, peroxidase; APX, ascorbate peroxidase; LAC, lacase; PAL, phenylalanine ammonia lyase; FDA, fluorescein diacetate hydrolase; HR, hydroxylamine reductase; β - Glu, β – glucosidase; SOD, superoxide dismutase; GLS, glutaminase; ASP, asparaginase; AMY; amylase; α-AMY, α-amylase; β-AMY, β-amylase; Cel, cellulase; Inv, invertase; ACP, acid phosphatase; NR, nitrate reductase; CAT, catalase; NUC, ribonuclease; Ure, urease; Prot, protease.

**Figure 10** and **Supplementary Table S4** presented the correlation analysis results between ginsenosides in leaves and soil properties. In G1, ginsenosides Rb3, Rh2, and Rc were most significantly affected by soil abiotic factors (**Figure 10A**), while PPO and GLS were the most considerable soil enzymes affecting ginsenoside accumulation (**Figure 10B**). In G2, ginsenosides Rb1, Rf, and Rd were most significantly affected by soil abiotic factors (**Figure 10C**), while AMY, HR, and Prot were the most considerable soil enzymes affecting ginsenoside accumulation (**Figure 10D**). In G3, ginsenosides Rb1, Rb2, and Re were most significantly affected by soil abiotic factors (**Figure 10E**), while trace elements such as T-Nd, T-Mn, T-Ce, and T-Y had the greatest impact on ginsenoside accumulation. AMY, PPO, and POD were the main soil enzymes that affect the accumulation of ginsenosides (**Figure 10F**).

#### 3.8.4 Correlation analysis between soil enzyme activity and abiotic factors

**Figure 11:**
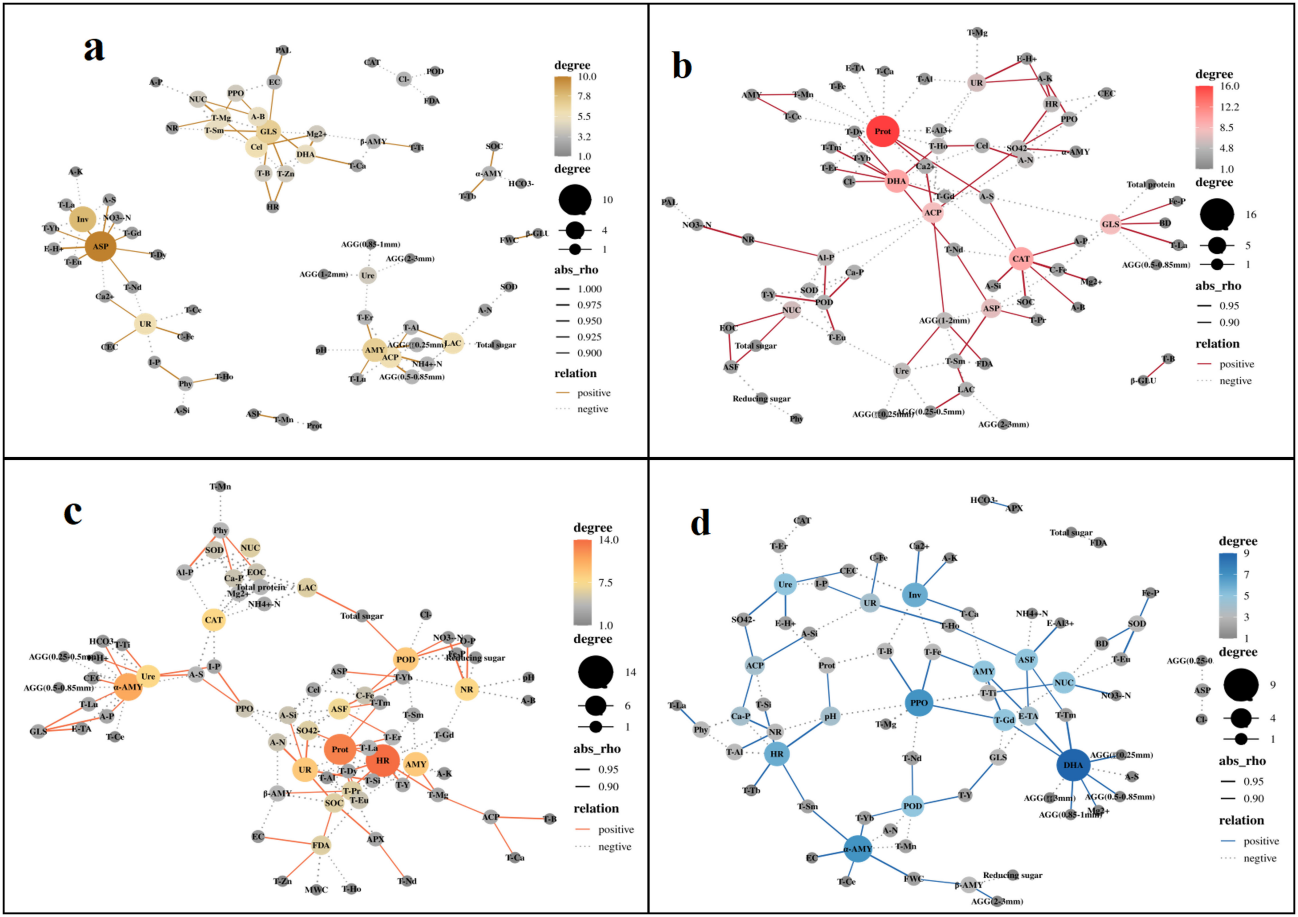
Correlation analysis between soil physicochemical properties and enzyme activity (**a**: G0; **b**: G1; **c**: G2; **d**: G3). Only results with *p<0.05* are displayed, with solid lines representing positive correlations and dashed lines representing negative correlations. The size and color of nodes are positively correlated with the number of connections, with the more connections, the larger and darker the nodes. BD, bulk density; FWC, field moisture capacity; MWC, mass moisture content; AGG, soil aggregates; EC, electrical conductivity; A-N, alkaline hydrolysis nitrogen; A-K, available potassium; Ca-P, calcium bound phosphorus; Fe-P, iron bound phosphorus; Al-P, aluminum bound phosphorus; O-P, occluded phosphorus; A-P, available phosphorus; I-P, inorganic phosphorus; SOC, Organic carbon; EOC, easily oxidizable organic carbon; E-TA, exchangeable total acid; E-H^+^, exchangeable hydrogen ion; E-Al^3+^, exchangeable aluminum ion; A-B, available boron; A-S, available sulfur; A-Si, available silicon; NO_3_^—^N, nitrate nitrogen; NH_4_^+^-N, ammonium nitrogen; C-Fe, complexed iron; Ca^2+^, calcium ions; Mg^2+^, magnesium ions; Cl^-^, chloride ions; SO_4_^2-^, sulfate ions; HCO_3_^-^, bicarbonate ion; CEC, cation exchange capacity; T-B, total boron; T-Mn, total manganese; T-Ti, total titanium; T-Ca, total calcium; T-Mg, total magnesium; T-Fe, total iron; T-Al, total aluminum; T-Si, total silicon; T-Zn, total zinc; T-Eu, total europium; T-La, total lanthanum; T-Ce, total cerium; T-Pr, total praseodymium; T-Nd, total neodymium; T-Sm, total samarium; T-Gd, total gadolinium; T-Tb, total terbium; T-Dy, total dysprosium; T-Ho, total holmium; T-Er, total erbium; T-Tm, total thulium; T-Yb, total ytterbium; T-Lu, total lutetium; T-Y, total yttrium; DHA, dehydrogenase; Phy, phytase; ASF, arylsulfatase; UR, uricase; PPO, polyphenol oxidase; POD, peroxidase; APX, ascorbate peroxidase; LAC, lacase; PAL, phenylalanine ammonia lyase; FDA, fluorescein diacetate hydrolase; HR, hydroxylamine reductase; β - Glu, β – glucosidase; SOD, superoxide dismutase; GLS, glutaminase; ASP, asparaginase; AMY; amylase; α-AMY, α-amylase; β-AMY, β-amylase; Cel, cellulase; Inv, invertase; ACP, acid phosphatase; NR, nitrate reductase; CAT, catalase; NUC, ribonuclease; Ure, urease; Prot, protease.

In the course of exploring the potential relationship between soil enzyme activity and abiotic factors, a correlation analysis based on Spearman test was conducted (**Supplementary Table S5**), and a correlation network diagram was constructed (**Figure 11**). ASP, Inv, and GLS were the core nodes in the G0 network (**Figure 11A**), Prot, DHA, ACP, CAT, and GLS were the core nodes in the G1 network (**Figure 11B**), HR, Prot, α-AMY, and POD were the core nodes in the G2 network (**Figure 11C**), and DHA, PPO, α-AMY, HR, and Inv were the core nodes in the G3 network (**Figure 11D**).

## 4 Discussion

### 4.1 Ginsenoside content of ginseng with different stem numbers

Ginsenosides are the most active chemical substances in the pharmacological activity of ginseng, so in this study, we used the content of ginsenosides as the evaluation standard for ginseng quality. September is the traditional harvesting period for ginseng, and the roots are the traditional medicinal parts of ginseng. We found that the content of most ginsenosides in MG was higher than that in G1 (**Figure 3D**), and PCA results showed a significant separation between MS and G1 (**Figures 6D, G**), indicating that MG had significantly higher pharmacological value than G1. Since ginseng can only be used for medicinal purposes at the age of 4 and above, although the ginseng in this study did not reach the age for medicinal use, we preliminarily determined that the cultivation of MG had practical significance. The results of this research were consistent with Zhang et al. (2012), who also reported that the total ginsenosides content of MG harvested at 3 years old was not significantly different from that of G1 harvested at 4 years old, which confirmed the high pharmacological value of MG.

Although leaves were not a traditional medicinal part of ginseng, the *Pharmacopoeia of the People’s Republic of China* has already included ginseng leaves as medicinal, so analyzing the content of active ingredients in ginseng leaves also has practical significance. According to the results of PCA (**Figures 6F, G**), the leaves of G1, G2, and G3 were significantly separated. The results of ginsenoside content (**Figure 3F**) showed that the pharmacological value of G1 leaves does not seem to lag behind that of MG. The large overlap of MG leaves may be one of the important reasons that hinder the maximization of photosynthesis. Many leaves that can undergo photosynthesis are wasted, resulting in a small difference in ginsenoside content compared to G1 (Zhang et al., 2012). However, due to the presence of a large number of leaves, MG still has a high economic value in terms of quantity alone.

### 4.2 Endogenous hormone content of ginseng with different stem numbers

Artificial induction and natural production are the two main reasons for the formation of MG. Artificial induction mainly involves removing ginseng buds from the base when they grow to the size of sorghum grains (Du, 1986); the excessive content of nutrients in soil and favorable ecological climate conditions are natural causes of MG production, and at this time, the endogenous hormone content of MG will also undergo significant changes compared to G1 (Zhao et al., 2020). According to local farmers, MG in the sampling area of this study is a non-human induced phenomenon, and G2 has the highest number. Therefore, the exploration of soil properties and endogenous hormone content in this study is meaningful.

The results of PCA showed a significant difference in endogenous hormone levels between G1 and MG (**Figure 6C**), indicating that hormone differences may be the cause of MG production. The salicylic acid content of MG was significantly higher than that of G1, while the brassinolide content of G1 was significantly higher than that of MG (**Table 1**). Salicylic acid is a major signaling regulator in plants, with effects on plant stress resistance and growth (Pieterse et al., 2012). Salicylic acid treatment increased the expression levels of endogenous phenylalanine ammonia lyase gene *PgPAL1* (Farh et al., 2020) and β -1,3-glucanase encoding gene *PqGlu-1* (Kiselev et al., 2006), as well as affect the accumulation of ginsenosides in *Panax ginseng* and *Panax quinquefolius* L (Kochan et al., 2019). Therefore, the higher content of ginsenosides in MG roots is associated with stress resistance and salicylic acid. Salicylic acid has been reported to play important roles in root hair formation (García-Sánchez et al., 2015), root elongation, lateral root formation (Kim et al., 2012), root wavy growth (Zhao et al., 2015), and root meristem formation (Pasternak et al., 2019) in *Arabidopsis thaliana*. Environmental stress stimulates the production of endogenous hormones and secondary metabolites (Li et al., 2022). For example, under high levels of *Ilyonectoria* sp. stimulation, the expression of ginsenoside synthase genes [farnesyl pyrophosphate synthase (*PgFPS*), squalene synthase (*PgSS1*), squalene epoxidase (*PgSE1*), and dammarenediol-II synthase (*PgDDS*)] in ginseng roots undergoes significant changes, thereby stimulating the production of ginsenosides; at the same time, the expression of the salicylic acid synthesis gene phenylalanine ammonia lyase gene (*PgPAL1*) increased synchronously to cope with biological stress (Bischoff Nunes and Goodwin, 2022). The production of ginsenosides changes the pharmacological value of ginseng, while the production of salicylic acid may alter the morphology of ginseng, such as the production of MG. The relative abundance of *Ilyonectoria* can be indirectly reflected by the enzymes produced by the *Ilyonectoria* species, including chitinase Prot, dextranase (Behdarvandi et al., 2023), pectinase, Cel (Rahman and Punja, 2005), PAL, and PPO (Farh et al., 2018). Since this study did not measure changes in rhizosphere fungi, changes in soil enzyme activity might indirectly reflect this change. We observed that the activities of Prot, Cel, PPO, and PAL in the rhizosphere soil of G3 were higher than those in G1 and G2, and the activities of these enzymes in ginseng rhizosphere soil were also higher than those in bulk soil (G0), which might confirm our hypothesis (**Figure 2**). In summary, we suggested that future research could pay more attention to the microbial dynamics in the rhizosphere soil of MG. Microorganisms may play a considerable role in the morphological production of MG, which provides many benefits for its cultivation.

Brassinolide is a type of plant specific steroid hormone that can control the opening and closing of stomata in plant leaves to enhance photosynthesis and promote the synthesis of photosynthetic products (Li et al., 2020). Brassinolide also has the ability of increasing the germination rate of plant seeds, promoting root growth, and enhancing the activity of antioxidant enzymes in plants to alleviate the toxic effects of salt stress on plants (Otie et al., 2021). Unlike salicylic acid, although brassinolide also plays a promoting role in root cell growth and division, more research has focused on enhancing plant photosynthesis and resistance to abiotic stress (heavy metals, salt, low temperature, and drought stress). Representative plants include rice (Nakagawa et al., 2021), grapes (Zhou et al., 2024), tomatoes (Mumtaz et al., 2020), and rapeseed (Xiong et al., 2021). We found that the content of most ginsenosides in the leaves of G1 was higher (**Figure 3F**), and the ternary plot showed that the leaves of G1 were enriched with more types of ginsenosides (**Figure 5C**). At the same time, the brassinolide of G1 was significantly higher than that of G2 and G3 (**Table 1**), which might explain why brassinolide plays a more important role in promoting photosynthesis in MG and G1. Therefore, we speculated that brassinolide might have a greater impact on metabolic function, although some effects had also been reported in the differentiation of rhizome, which required further research to confirm. Prusinkiewicz et al. (2009) argued that appropriate hormones were necessary for plant growth, and above or below this threshold may lead to changes in plant phenotype, such as the appearance of multiple branching. We speculated that the significant differences in the content of salicylic acid and brassinolide in MG compared to G1 may be one of the causes of ginseng multi stem phenomenon, which was a future research direction because MG had higher economic value than G1.

### 4.3 Effects of soil properties on ginsenoside accumulation

Previous studies have confirmed that soil elements (Ma et al., 2021; Zhu et al., 2021) and enzyme activity (Behdarvandi et al., 2023; Chu et al., 2023) have a significant impact on the accumulation of ginsenosides. In this study, we measured the large, medium, and trace elements (including rare earth elements) required by plants in soil. The results showed that Al-P, AGG (2–3 mm), T-Pr, and E-Al^3+^ in soil were the main factors causing differences in G0, G1, G2, and G3. Aluminum bound phosphorus (Al-P) belongs to the inorganic phosphorus (I-P) component and needs to be decomposed by microorganisms into available phosphorus (A-P) in order to be absorbed and utilized by ginseng (Sang et al., 2022). Phosphorus solubilizing microorganisms play a major role in this process, and the common ways of phosphorus solubilization are (1) biological phosphorus solubilization: microorganisms secrete phosphorus cycling enzymes (phosphatases, phytases, ribonucleases), which use the function of enzymes to decompose substrates to increase the availability of phosphorus in soil (Grierson and Comerford, 2000; Manzoor et al., 2017); (2) chemical phosphorus solubilization: phosphorus solubilizing microorganisms produce small molecule organic acid metabolites, which lower soil pH and dissolve mineral phosphates in the soil, thereby increasing phosphorus availability (Sang et al., 2022). At the bacterial genus level, *Bacillus*, *Micrococcus*, *Pseudomonas*, *Clromobacter*, *Burkholderia*, etc., and at the fungal genus level, *Penicillium*, *Enterobacter*, *Rhizopus*, *Serratia*, *Aspergillus*, etc. all have phosphorus solubilizing functions (Bamagoos et al., 2021). When Al is present in high concentrations, it can inhibit crop growth. Specifically, the forms of Al in soil include exchangeable aluminum ions (E-Al^3+^), monomeric hydroxyl aluminum (Hy-Al), acid soluble inorganic aluminum (Ac-Al), and aluminum humate (Hu-Al). Among them, E-Al^3+^ is the aluminum form with the highest risk of aluminum toxicity (You et al., 2015). Aluminum may play a more critical role in the formation and differentiation of G1 and MG quality.

Soil enzymes are bioactive substances in soil that react quickly to external disturbances and are vital indicators for evaluating soil fertility and environmental quality (Dindar et al., 2015). The results of this study showed that the enzyme activity of most of the soil in G3 was significantly higher than that of other groups, indicating that G3 had a stronger demand for nutrients and required higher enzyme activity to promote the formation of available nutrients, which was closely related to microbial activity. Although ginseng roots also release enzymes into the soil, most of the enzymes in the soil come from soil microorganisms. For example, xylanase and glycoside hydrolase are derived from *Mortierella* (Fang et al., 2016), *Acremonia* (Watanabe et al., 2014), *Roseiflexus* (Lee et al., 2018) and *Rubrobacter* (Ceballos et al., 2017), these enzymes play a role in the decomposition of cellulose, hemicellulose, or lignin in soil. Random forest analysis showed that FDA was a characteristic enzyme for soil differences among different groups (**Figure 7B**), and FDA activity reflected well the microbial activity in soil, changes in soil quality, and the rate of organic matter transformation in ecosystems (Xiang et al., 2022). Therefore, we believed that the enzymes produced by microorganisms were the main driving force for promoting soil nutrient cycling and ginsenoside accumulation, although due to space limitations, this study did not measure soil microorganisms.

**Figure 12:**
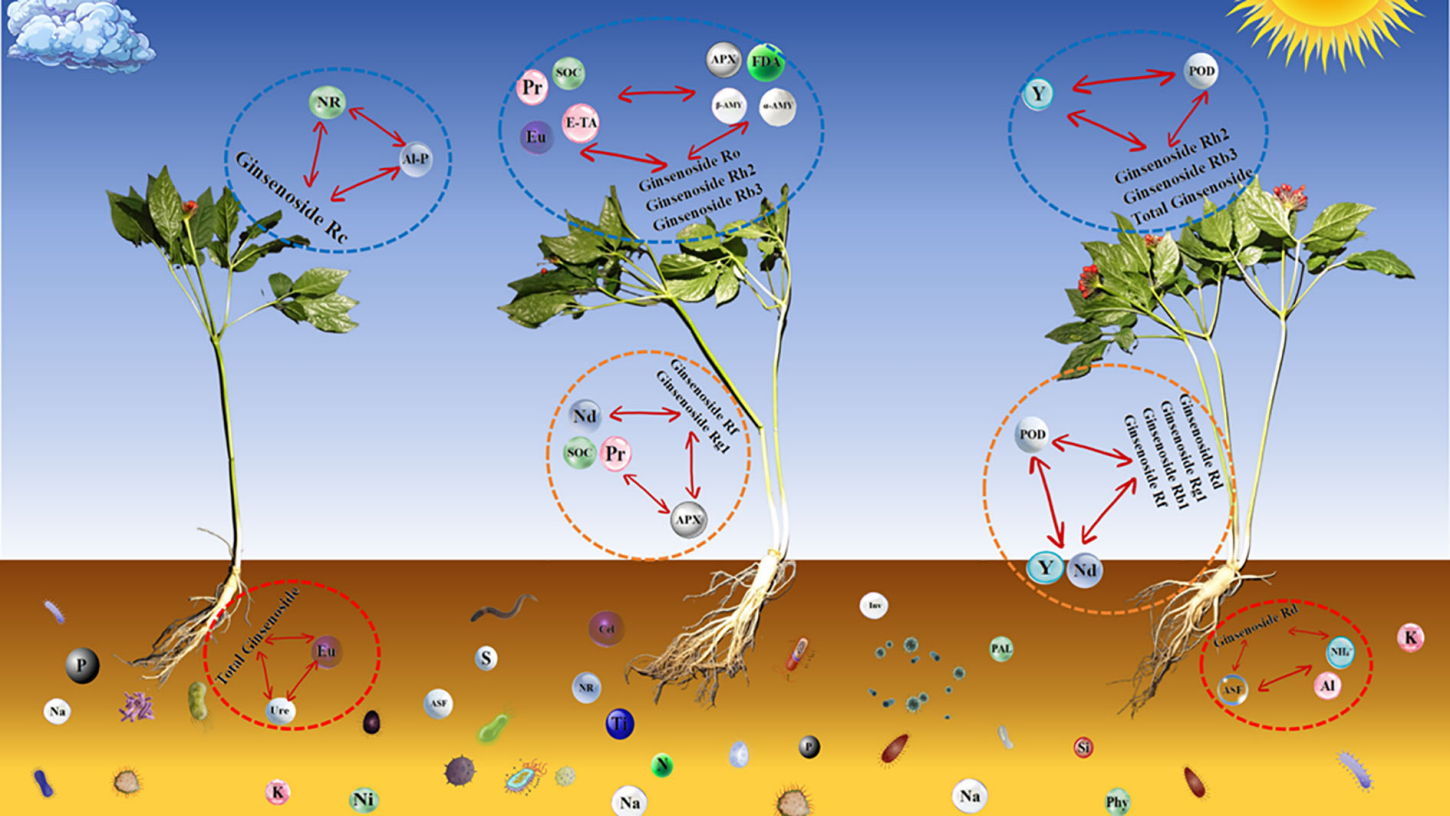
Potential regulatory network of “soil abiotic factors-ginsenosides-soil enzymes”.

According to the correlation analysis, we found that although there were many non characteristic soil factors such as A-N, A-P, A-K, UR, NUC, HR, and ginsenosides that have significant correlations, we expected to find some characteristic associations from them. Therefore, we focused on observing the characteristic factors that ranked high in random forest analysis (**Figure 7**) and presented the results in **Supplementary Table S6**. Based on the results in **Supplementary Table S6**, we plotted the regulatory network of “soil abiotic factors-ginsenosides-soil enzymes” (**Figure 12**). Soil enzymes mainly come from rhizosphere microorganisms, so clarifying the significant role of certain enzymes can reflect the primary role of certain groups of microorganisms in the accumulation of ginsenosides. In terms of this study, *Gemmata*, *Longispora*, *Conexibacter*, *Prausurella*, and *Sterolibacterium* have the ability to produce NR (Jara-Servin et al., 2024), while *Morganella* and *Providencia* have the ability to produce Ure and produce biogenic amines for neutralizing acidic substances in soil (Shumo et al., 2021). ASF is widely present in *Planctomycetes* (Glöckner et al., 2003), and at the same time, the *M1803* strain in *WD2101_soil_group* also exhibits sulfatase with the ability to degrade sulfated sugar polymers (Dedysh et al., 2021). Some members of the *Bacillus* genus have phosphate solubilizing properties similar to phosphatase or phytase, such as *Bacillus notoginsengsoli* which has positive reactions for phosphatase, CAT, and Ure (Zhang et al., 2017), and *Bacillus licheniformis* DSM-13 which has been reported to have the potential for non natural biosynthesis of ginsenosides (Dai et al., 2018). In summary, we speculated that these factors played a considerable role in the accumulation of ginsenosides, which may have significance in future research.

## 5 Conclusion

The results of this study indicated that compared with G1, the roots and leaves of MG had higher pharmacological activity and economic value, which was of practical significance for the study of MG. The analysis of rhizosphere and non-rhizosphere soil properties showed that the soil enzyme activity in G3 was significantly higher than that in other groups. At the same time, FDA, as a characteristic factor for the differences in samples between different groups, reflected the differences in microbial activity in different soils. Thus, subsequent research should focus on observing the seasonal changes in the rhizosphere microecology of MG and conducting correlation analysis with the quality indicators of ginseng to further clarify the mechanism of MG quality formation. Among endogenous hormones, we found that salicylic acid and brassinolide were the main causes of MG formation, we suggest considering stimulating the production of these hormones to promote the multi stem transformation of ginseng, enhance its medicinal value, and increase farmers’ income. Y and Nd had significant effects on the accumulation of ginsenosides in G3 among rare earth elements, future research needs to consider the role of these rare earth elements as abiotic factors in the formation of MG morphology and pharmacological value, as well as the potential role of soil microorganisms in the formation of MG morphology and quality. Besides, we have also constructed a regulatory network of “soil abiotic factors-ginsenosides-soil enzymes”, after determining the potential effects of these abiotic factors in the laboratory, farmers can cultivate high pharmacological activity MG production by measuring these indicators. The results have improved our understanding of MG and contributed to the better development of the MG industry.

## Data Availability

The raw data supporting the conclusions of this article will be made available by the authors, without undue reservation.
